# Suppressed intestinal secondary bile acids in moxifloxacin-induced hyperglycemia: studies in normal and diabetic GK rats

**DOI:** 10.3389/fphar.2025.1569856

**Published:** 2025-04-04

**Authors:** Yewen Sun, Yuchen Qu, Zhuan Yang, Bo Lv, Guanjun Wang, Kai Fan, Yuyuan Wang, Jie Pan, Ziyan Du, Yunli Yu

**Affiliations:** ^1^ Department of Pharmacy, The Second Affiliated Hospital of Soochow University, Suzhou, China; ^2^ College of Pharmaceutical Science, Soochow University, Suzhou, China; ^3^ School of Pharmacy, Nanjing Medical University, Nanjing, China; ^4^ Department of Respiration Medicine, The Second Affiliated Hospital of Soochow University, Suzhou, China

**Keywords:** bile acid, moxifloxacin, hyperglycemia, gut microbiota, GLP-1

## Abstract

**Objective:**

Moxifloxacin (MFLX) frequently induces dysglycemia when used in the treatment of infectious diseases, particularly in patients with diabetes. However, the mechanism through which MFLX affects host glucose metabolism remains unclear. This study aimed to investigate the possible mechanism underlying MFLX-induced hyperglycemia.

**Methods:**

In this study, we investigated the short-term (3 days) and long-term (14 days) effects of MFLX on glucose metabolism in normal and type 2 diabetic GK rats. After oral administration of 40 mg/kg of MFLX, blood glucose, insulin, GLP-1, and fibroblast growth factor 15 (FGF15) levels in the blood of rats, as well as bile acids in both blood and feces, and gut microbiota, were examined. Liver and ileum tissues were promptly harvested for detecting the expression of hepatic 7α-hydroxylase (CYP7A1) and intestinal Takeda G-protein-coupled receptor 5 (TGR5) and farnesoid X receptor (FXR). In addition, we explored the effect of secondary bile acids (SBAs) on GLP-1 secretion in NCI-H716 cells, and observed the direct effect of MFLX on the expression of CYP7A1 in HepG2 cells and TGR5, FXR in NCI-H716 cells.

**Results:**

It was demonstrated that MFLX induced hyperglycemia in diabetic rats, with a more pronounced reduction in serum insulin, GLP-1, and FGF15 levels than observed in normal rats. Gut microbiota associated with SBAs metabolism were significantly reduced, leading to decreased intestinal deoxycholic acid (DCA) and lithocholic acid (LCA). *In vitro* studies revealed that DCA and LCA (25 μM, 50 μM, and 100 μM) promoted GLP-1 secretion in a concentration-dependent manner in NCI-H716 cells. Meanwhile, we observed that the expression of intestinal TGR5 and FXR significantly downregulated, whereas CYP7A1 expression in liver was increased in GK rats after MFLX treatment. MFLX itself (0.1 μM, 1 μM, and 10 μM) did not directly altered TGR5 or FXR expressions in NCI-H716 cells, nor did it alter CYP7A1 expression in HepG2 cells, which indicated that the impact of MFLX on glucose metabolism was primarily induced by changes in bile acids metabolism resulting from alterations in the gut microbiota.

**Conclusion:**

Our studies showed MFLX more likely to cause hyperglycemia when used in diabetic states and highlighted the critical role of gut microbiota-SBAs-TGR5/FXR pathway in MFLX-induced hyperglycemia.

## 1 Introduction

Fluoroquinolones are widely prescribed to treat a range of bacterial infections, including urinary tract infections, skin and soft tissue infections, as well as nosocomial and community-acquired pneumonia (Eckmann and Tulkens, 2021; ten Doesschate et al., 2022; Belforti et al., 2016). However, the increased use of these agents has been associated with rare yet clinically significant adverse events, including hyperglycemia and hypoglycemia (El Ghandour and Azar, 2015; Granados et al., 2018; Iguchi et al., 2020), particularly in patients with or at risk of diabetes (Singh et al., 2023; Althaqafi et al., 2021). A prospective observational study indicated that dysglycemia was more frequently encountered with moxifloxacin (MFLX) use, with hyperglycemia occurring more common than hypoglycemia; the incidence of hyperglycemia associated with MFLX was reported to be 33.3% (Kabbara et al., 2015). Furthermore, a nationwide diabetes cohort study discovered that the absolute risk of hyperglycemia per 1,000 individuals was 6.9 for those taking MFLX. Patients taking MFLX faced a significantly higher risk of dysglycemia than those receiving other fluoroquinolones (Althaqafi et al., 2021; Chou et al., 2013). Some studies have proposed several possible mechanisms by which MFLX causes dysglycemia, including stimulating histamine secretion (Ishiwata et al., 2013), inhibition of Kv11.1 channels (Juhl et al., 2023) or HERG protein (Qiu et al., 2016) in pancreatic β-cells, which impaired membrane depolarization and insulin secretion. However, it remains unclear why the extent of glucose disturbances caused by MFLX varies under different physiological and pathological conditions. In this study, we provided novel insights into MFLX-induced glucose metabolism dysregulation through the gut microbiota-bile acid (BA) metabolism axis.

BAs play a crucial role in lipid, glucose, and energy metabolism. As antimicrobial drugs, fluoroquinolones can alter the composition and activity of intestinal microbiota, with changes in the gut microbial community impacting BAs profiles in plasma and feces (Zhan et al., 2024; Behr et al., 2019; Beekman et al., 2024). Primary bile acids (PBAs), cholic acid (CA) and chenodeoxycholic acid (CDCA), are synthesized from cholesterol in hepatocytes via 7α-hydroxylase (CYP7A1) and secreted into the intestine as glycine- and taurine-conjugated forms (Jia et al., 2018). Gut microbiota play crucial roles in maintaining BAs homeostasis *in vivo*. In the gut, PBAs are further metabolized by microbial enzymes into unconjugated secondary bile acids (SBAs), such as deoxycholic acid (DCA) and lithocholic acid (LCA) (Chiang, 2017; Kiriyama and Nochi, 2023). Given this interaction, we aimed to investigate the potential link between MFLX-induced dysglycemia and its effect on gut microbial-BAs metabolism.

SBAs play a pivotal role in regulating energy metabolism by binding to nuclear receptors, including Takeda G-protein-coupled receptor 5 (TGR5) and farnesoid X receptor (FXR) (Perino et al., 2021; Fiorucci et al., 2021; Cariello et al., 2018; Pathak et al., 2018; Gonzalez et al., 2017; Münzker et al., 2022). Specific BAs (LCA > DCA > CDCA > CA) activates intestinal TGR5 receptor (Castellanos-Jankiewicz et al., 2021; Gao et al., 2022; Jiao et al., 2022), leading to increased intracellular cAMP synthesis and Ca2^+^ concentration, which promotes the release of GLP-1 from intestinal endocrine cells through the exchange protein directly activated by cAMP (Epac)-mediated signaling pathway (Zeng et al., 2024; Qi et al., 2021; Kim et al., 2014). Conversely, activation of intestinal FXR by BAs (CDCA > CA > LCA > DCA) in the intestinal epithelium induces transcription of fibroblast growth factor 19 (FGF19; FGF15 in rodents), a circulating endocrine factor involved in energy metabolism. Once secreted, FGF15/19 enters the portal circulation and travels to the liver, and suppresses CYP7A1 transcription through an SHP-dependent manner, ultimately reducing BAs synthesis (Han et al., 2023; Katafuchi and Makishima, 2022; Ye et al., 2023). Despite these established mechanisms, it remains unclear whether MFLX-induced hyperglycemia correlated with BAs pathways.

In this study, we investigated the effects of MFLX on glucose metabolism in both normal and type 2 diabetic GK rats, a well-established, non-obese, spontaneous model of type 2 diabetes closely mimicking the pathophysiology of human diabetes (Akash et al., 2013), aiming to uncover potential mechanisms underlying MFLX-induced dysglycemia.

## 2 Materials and methods

### 2.1 Chemicals and reagents

Moxifloxacin (98%, Lot#: C15243309) were obtained from Shanghai Macklin Biochemical Co., Ltd. (Shanghai, China). CA, CDCA, ursodeoxycholic acid (UDCA), DCA, LCA, taurocholic acid (TCA), taurochenodeoxycholic acid (TCDCA), tauroursodeoxycholic acid (TUDCA), taurodeoxycholic acid (TDCA), taurolithocholic acid (TLCA), glycocholic acid (GCA), glycochenodeoxycholic acid (GCDCA), glycoursodeoxycholic acid (GUDCA), glycodeoxycholic acid (GDCA), α-muricholic acid (α-MCA), β-muricholic acid (β-MCA), tauro-α-muricholic acid (TαMCA), tauro-β-muricholic acid (TβMCA), and cholic acid-d4 (internal standard) were all procured from Sigma-Aldrich (St. Louis, MO, United States). Glycolithocholic acid (GLCA) was obtained from J&K Scientific (Beijing, China). All reagents and solvents, including HPLC-grade ammonium formate (≥99%), ammonium acetate, methanol, and acetonitrile, were sourced from Merck KGaA (Darmstadt, Germany).

### 2.2 Animals and treatment

Male 9-week-old wistar rats and GK rats were obtained from Shanghai SLAC Laboratory Animal Co., Ltd. (Shanghai, China). Following a 1-week acclimation period under controlled conditions (12-h light/dark cycle at 21°C ± 2°C) with *ad libitum* access to food and water, the Wistar rats were randomly assigned to either the CT group or the CT + MFLX group, and the GK rats were assigned to the GK group or the GK + MFLX group, each group comprising 5 animals balanced by body weight. Wistar rats received a standard chow diet, whereas GK rats were provided a high-fat diet (HFD). The HFD provided to GK rats consisted of 20% crude protein, 40% fat, and 40% carbohydrates, which was obtained from Shanghai SLAC Laboratory Animal Co., Ltd. Additionally, the CT + MFLX and GK + MFLX groups were administered MFLX at a dose of 40 mg/kg via gavage for 3 and 14 days, respectively. The CT and GK groups received a 0.25% CMC-Na solution as a vehicle control. All animal experiments were approved by the Animal Ethics Committee of Soochow University (SUDA20241121A03).

### 2.3 Serum and tissue sample collection

After treatment with MFLX for 3 and 14 days, rats were fasted overnight for 12 h. Fecal samples were collected and stored at −80°C for subsequent analysis. Following this, a glucose dose of 2.5 g/kg was administered via gavage, and blood samples were collected from the orbit at baseline (0 min), and at 15 min and 30 min post-glucose administration. Then the serum samples were collected by centrifugation at 4,000 rpm for 10 min at 4°C and stored at −80°C for future analysis.

After 2 h of glucose loading, the rats were anesthetized with an intraperitoneal injection of sodium pentobarbital (60 mg/kg). Blood samples were collected via arterial cannulation, and ileum and liver tissues were excised. Serum samples were prepared and stored following previously described protocol, and all collected tissue samples were stored at −80°C for subsequent testing.

### 2.4 16S rRNA sequencing analysis

Total genomic DNA from the intestinal contents was extracted using the HiPure Stool DNA Kit (Megan, Guangzhou, China) following the manufacturer’s protocols. DNA concentration was measured using the Equalbit dsDNA HS Assay Kit (Novizan, Nanjing China). The NGS library preparation and Illumina sequencing were performed by GENEWIZ, Inc. (Suzhou, China). Approximately 20–50 ng of DNA was used to generate amplicons. The V3 and V4 hypervariable microbial 16S rRNA regions were amplified by PCR using a panel of proprietary primers designed by GENEWIZ. Subsequently, the PCR product from 16S rRNA was spliced by PCR to the end of the 16S rRNA product with an Index for NGS sequencing. The final PCR product libraries were analyzed by 1.5% agarose gel electrophoresis to detect the presence of the target fragment at 600 bp. The obtained sequencing library was subsequently purified with magnetic beads, followed by library quality control checks using a microplate reader and agarose gel electrophoresis. Library concentration was measured by enzyme assay (Tecan, Infinite 200 Pro) and sequencing was performed on PE250/FE300 double-end sequencing according to the instructions of the Illumina MiSeq/Novaseq (Illumina, San Diego, CA, United States) and PE250/FE300 double-end sequencing were performed according to Illumina’s instructions.

Double-end sequencing of positive and negative reads the first of the two joining together to filter joining together the results contained in the sequence of N, retains the sequence length is larger than 200 bp sequence. After quality filter, purify chimeric sequences, the resulting sequence for OTU clustering, use VSEARCH clustering (1.9.6) sequence (sequence similarity is set to 97%), than the 16 s rRNA reference database is Silva, 138. Then use RDP classifier (Ribosomal Database Program) bayesian algorithm of OTU species taxonomy analysis representative sequences, and under different species classification level statistics community composition of each sample.

### 2.5 Determination of blood glucose

Blood glucose were measured by the glucose oxidase method following the manufacturer’s instructions provided with the kits from Nanjing Jiancheng Bioengineering Institute.

### 2.6 Enzyme-linked immunosorbent assays

Serum insulin concentration was measured using the Mercodia Rat Insulin ELISA kit (Mercodia, Cat#10-1250-01). Serum active GLP-1 levels were assessed with the GLP1 (active) assay kit (Millipore, Cat#EGLP-35K), and serum FGF15 levels were determinated using the Rat Fibroblast Growth Factor 15 (FGF15) ELISA Kit (SAB, Cat#EK12289). All assays were performed according to the manufacturers’ protocols to ensure accuracy and consistency in the measurements.

### 2.7 BAs analysis

The separation of the target compounds was performed on a on Waters Xterra RP18 column (4.6 mm × 150 mm i.d., 5 μm, Waters Corp.) at 40°C with a Phenomenex Security GuardTM C18 Pre-column (4 mm × 3.0 mm) using a Aglient 1200 HPLC system. The mobile phase consisted of 10 mM ammonium acetate (A) and acetonitrile (B). The mobile phase flow rate was 0.8 mL/min and an injection volume of 20 μL. The elution gradient started with 30% phase B for 16 min, increased to 90% from 16 to 18 min, held at 90% for 5 min, and then decreased back to 30% at 23.5 min, followed by a 6.5-min equilibration at 30%.

Mass spectrometric detection was conducted in negative ion mode on an API-4000 Triple Quadrupole Tandem Mass Spectrometer (Applied Biosystem Sciex, United States). The optimal parameters were as follows: electrospray voltage: −4200 V, crash gas:4 psi, curtain gas: 30 psi, atomizing gas: 50 psi, auxiliary gas: 50 psi, ion source temperature: 500°C. Multiple reaction monitoring (MRM) mode was used for quantitative analysis. And the retention time and mass spectrometric parameters of 19 BAs were listed in Supplementary Table S1. All MS spectra were acquired and analyzed using the AB Sciex API 4000 system.

### 2.8 RNA extraction and real-time PCR

Intestinal mucosa scraped from the ileum and liver tissues were frozen in liquid nitrogen and stored at −80°C. Total RNA was extracted from the frozen tissues using a standard phenol-chloroform extraction with Trizol reagent. Subsequently, cDNA was synthesized from 2 μg of total RNA using the Reverse Transcription Kit (ABM, Cat#G490). Real-time quantitative PCR was conducted on an Applied Biosystems QuantStudio Dx platform (Thermo Fisher Scientific, United States) utilizing universal SYBR Green (APE × BIO, United States) as the fluorescent dye. Primer sequences for real-time PCR are detailed in Supplementary Table S2.

### 2.9 Western blot analysis

Total protein was extracted from ileum and liver samples (n = 3 per group) using RIPA buffer, and these extracts were utilized to assess the expression levels of FXR, TGR5, and CYP7A1 protein. Protein concentration was determined by the BCA method (Biosharp, China). Proteins were subsequently denatured and separated by gel electrophoresis, followed by wet-transfer to a membrane. After blocking the membrane for 2 h at room temperature, primary antibodies were applied at the following dilutions: FXR (1:1,000; Proteintech, China), TGR5 (1:500; Abmart, China), CYP7A1 (1:500; Proteintech, China), and β-actin (1:2000; Proteintech, China). Membranes were incubated with the primary antibodies overnight at 4°C.

The following day, membranes were washed three times with TBST (Sangon Biotech, Shanghai, China) for 10 min each. Secondary antibodies (1:10,000; Proteintech, China) were then applied for 1 h at room temperature. After further washing, ECL reagent was used for chemiluminescent detection of protein bands, which were subsequently quantified using ImageJ software.

### 2.10 Cell lines

Human NCI-H716 cells and HepG2 cells were obtained from National Collection of Authenticated Cell Cultures (Shanghai, China). NCI-H716 cells were cultured in RPMI 1640 medium (Gibco, United States), supplemented with 10% fetal bovine serum (FBS), 100 IU/mL penicillin, and 100 mg/mL streptomycin. To induce endocrine differentiation, cells were seeded in dishes pre-coated with Matrigel (Becton, Dickinson and Company, United States) and maintained in high-glucose DMEM (Gibco, United States) containing 10% FBS, 100 IU/mL penicillin, and 100 mg/mL streptomycin. HepG2 cells were cultured in DMEM supplemented with 10% FBS, 100 IU/mL penicillin, and 100 mg/mL streptomycin.

### 2.11 Effect of SBAs on GLP-1 secretion

Human enteroendocrine NCI-H716 cells were maintained in suspension culture, and differentiation was induced following the protocol described in a previous study. Two days prior to experimentation, 1.5 × 10^6^ cells were seeded into 24-well culture plates pre-coated with Matrigel and containing high-glucose DMEM supplemented with 10%FBS, 100 IU/mL penicillin and 100 mg/mL streptomycin. On the day of experimental day, the medium was replaced with Krebs-Ringer bicarbonate (KRB) buffer, supplemented with DCA and LCA at concentrations of 25 μM, 50 μM, and 100 μM. After a 2-h incubation at 37°C, supernatants were collected, with phenylmethylsulfonyl fluoride added at 50 mg/mL to inhibit protease activity, and samples were stored at −80°C for analysis. GLP-1 levels were quantified using a GLP-1 active ELISA kit according to the manufacturer’s instructions.

### 2.12 TGR5 and FXR expression in NCI-H716 and CYP7A1 expression in HepG2 cells

Two days prior to the experiments, 2.5 × 10^5^ NCI-H716 cells per well were seeded into 6-well culture plates pre-coated with Matrigel, containing high glucose DMEM supplemented with 10%FBS, 100 IU/mL penicillin, and 100 mg/mL streptomycin. On the day of the experiment, the medium was replaced with fresh medium containing MFLX at concentrations of 0.1 μM, 1 μM, and 10 μM. After a 24-h incubation at 37°C, cells were collected for protein and RNA extraction and stored at −80°C for Western blotting and RT-QPCR analysis to assess TGR5 and FXR expression.

For the HepG2 cells, 2.5 × 10^5^ HepG2 cells per well were seeded into 6-well culture plates. Upon reaching 90% confluence, the medium was replaced with fresh medium containing MFLX at 0.1 μM, 1 μM, 10 μM concentrations. After a 24-h incubation at 37°C, cells were collected for protein and stored at −80°C for Western blotting to determine CYP7A1 expression levels.

### 2.13 Data analysis

Statistical analysis was performed using GraphPad Prism software, version 9.3.0 (GraphPad Software, La Jolla, CA, United States). Experimental data were presented as the mean ± standard deviation (SD). Comparisons were performed using unpaired two-tailed Student’s t-test and one-way ANOVA followed by Tukey’s multiple comparison *post hoc* test. We conducted an analysis of intestinal BAs and microbiota using a linear regression model incorporating interaction terms.

## 3 Results

### 3.1 MFLX-induced hyperglycemia and changes in insulin, GLP-1 and FGF15 levels in normal and GK rats

**FIGURE 1:**
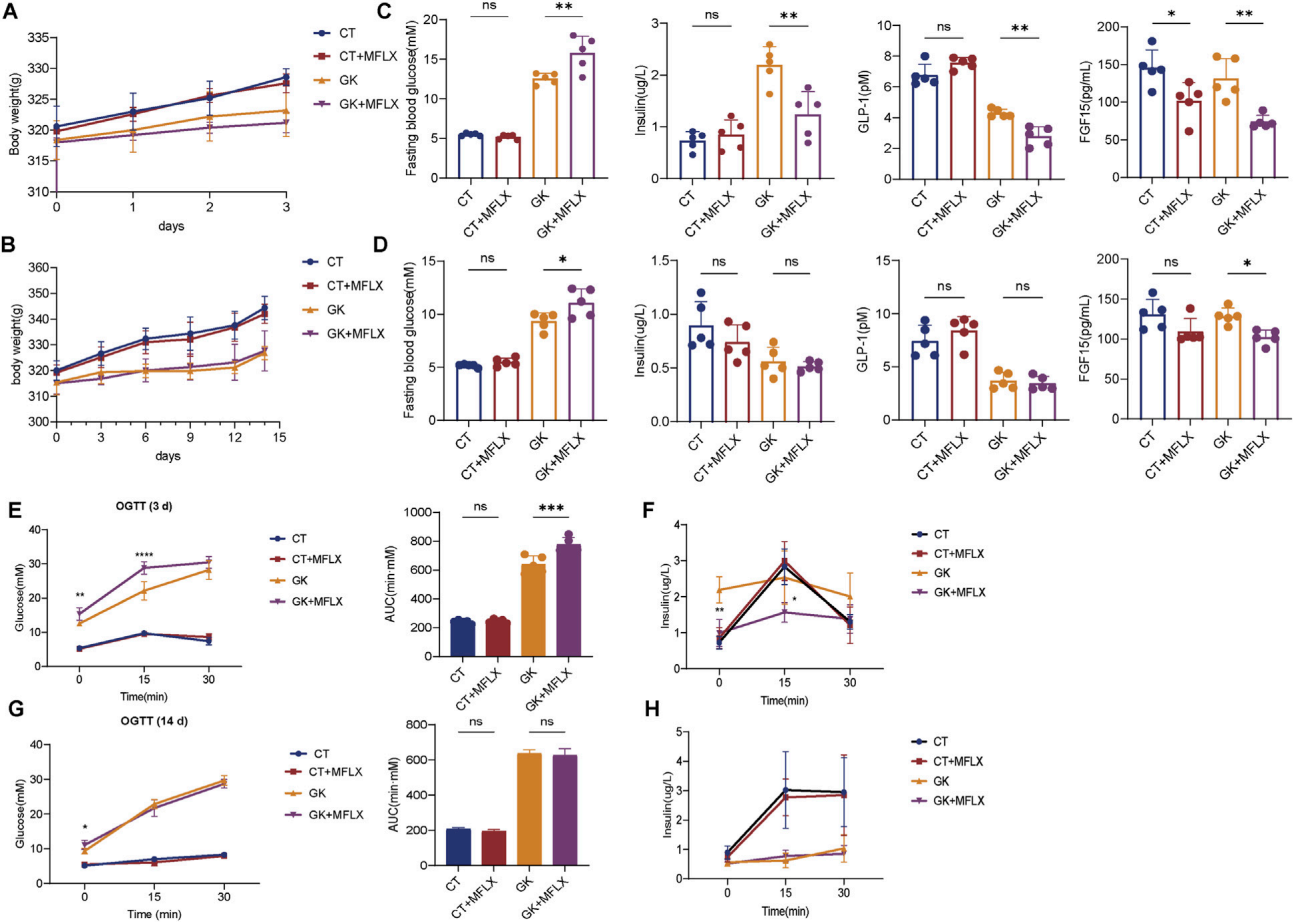
The effect of MFLX on blood glucose, insulin, GLP-1 and FGF15 levels in normal and GK rats. **(A, B)** The body weight of rats after MFLX treatment for 3 days **(A)** and 14 days **(B)**; **(C, D)** Changes in blood glucose, insulin, GLP-1, and FGF15 levels following MFLX administration for 3 days **(C)** and 14 days **(D)**; **(E, F)** Oral glucose tolerance test (OGTT) results **(E)** and glucose-induced insulin levels **(F)** after 3 days of administration; **(G, H)** OGTT **(G)** and glucose-induced insulin levels **(H)** after 14 days of MFLX administration. Data represent n = 5 per group. Statistical significance was determined using a two-tailed Student’s t-test, with *p < 0.05 and **p < 0.01. All data are presented as the mean ± SD.

It was showed that body weight of the CT rats increased from approximately 320.8 g–328.6 g (2.4%↑) over 3 days (Figure 1A), while the GK rats only grew from 318.4 g to 323.2 g (1.5%↑). Over 14 days (Figure 1B), body weight of the CT rats rose from 320 g to 344.4 g (7.6%↑), whereas the GK rats increased from 315.4 g to 326.8 g (3.6%↑). GK rats had a lower body weight than normal rats and exhibited slower weight gain. The results consistented with prior reports that adult GK rats exhibit 10%–30% lower body weight compared to normal Wistar rats (Akash et al., 2013). There was no significant effect on the body weight of normal rats or GK rats after MFLX treatment for 3 and 14 days (Figures 1A, B). After 3 and 14 days of MFLX administration, normal rats exhibited no significant changes in fasting blood glucose (FBG), insulin, or GLP-1 levels (Figures 1C, D), although FGF15 levels were significantly reduced following 3 days of treatment (Figure 1C). In contrast, GK rats showed a marked increase in FBG levels, particularly after 3 days of MFLX treatment, alongside a significant decrease in fasting insulin, GLP-1 and FGF15 levels (Figure 1C). Concurrently, the area under the curve (AUC) for the oral glucose tolerance test (OGTT) was significantly elevated (Figure 1E), and insulin secretion levels were reduced (Figure 1F). Following 14 days of MFLX treatment in GK rats, insulin and GLP-1 levels remained unaltered, whereas FGF15 levels continued to be reduced (Figure 1D). Additionally, no changes were observed in the AUC of the OGTT (Figure 1G) or in insulin secretion levels (Figure 1H).

### 3.2 Dynamic change of BAs profiles in serum and feces

**FIGURE 2:**
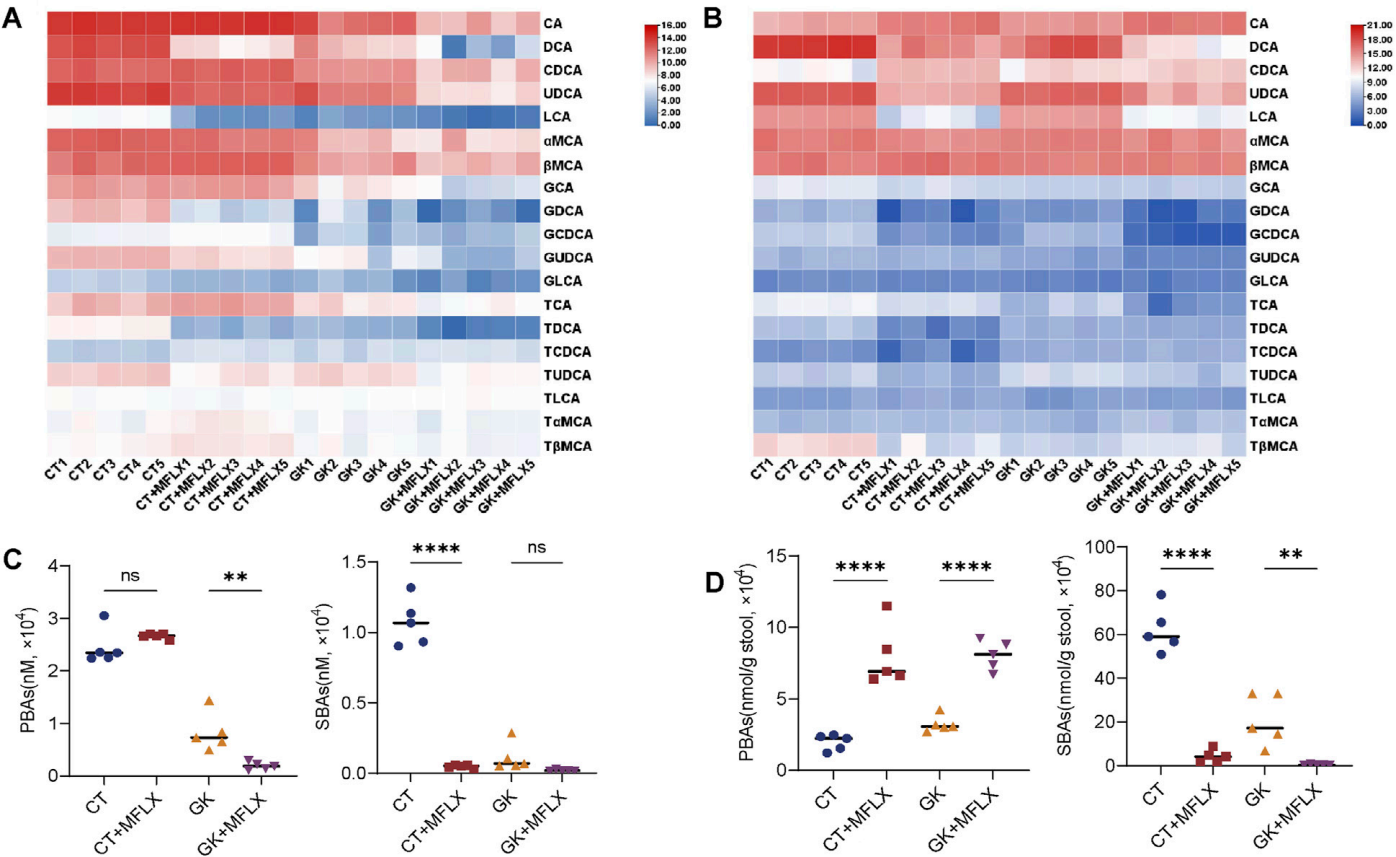
Dynamic change of BAs profile in serum and feces after MFLX treatment for 3 days. **(A, B)** Heatmaps showing 19 BAs in serum **(A)** and feces **(B)** after 3 days of MFLX treatment; **(C, D)** PBAs and SBAs in serum **(C)** and feces **(D)** following 3 days of MFLX treatment. Data are expressed as mean ± SD (n = 5). *P < 0.05, **P < 0.01, ***P < 0.001, ****P < 0.0001.

**FIGURE 3:**
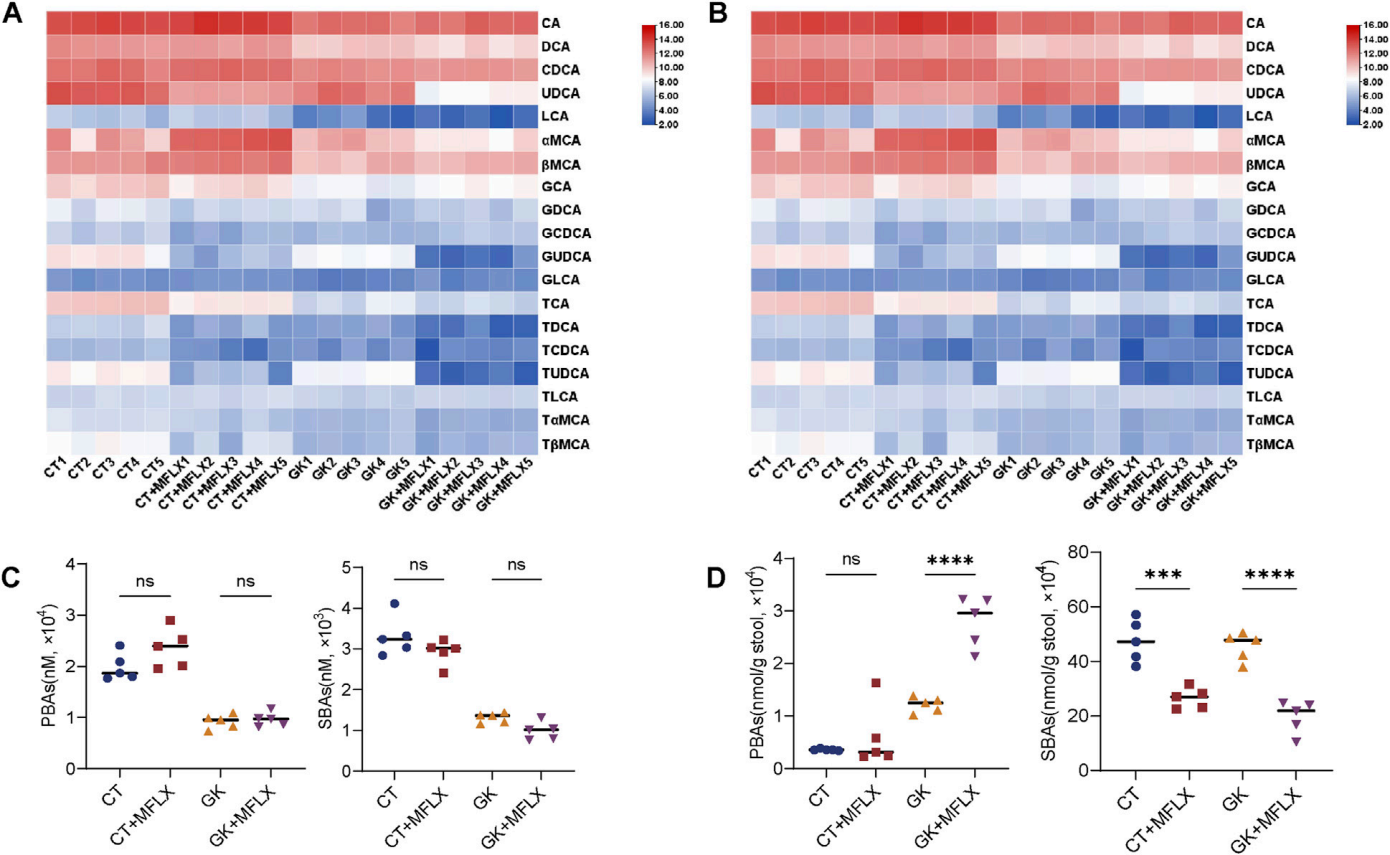
Dynamic change of BAs profile in serum and feces after MFLX treatment for 14 days. **(A, B)** Heatmaps showing 19 BAs in serum **(A)** and feces **(B)** after 14 days of MFLX treatment; **(C, D)** Levels of PBAs and SBAs in serum **(C)** and feces **(D)** after 14 days of MFLX treatment. Data are expressed as mean ± SD (n = 5). *P < 0.05, **P < 0.01, ***P < 0.001, ****P < 0.0001.

Heatmaps were generated to illustrate the effects of MFLX on serum and fecal BAs profiles in normal and GK rats (Figures 2A, B). In serum, total BAs were decreased in both CT + MFLX group (28%↓ compared with CT group) and GK + MFLX group (75%↓ compared with GK group) after 3 days treatment of MFLX (Supplementary Figure S1A). After 14 days, a slight upward trend of total BAs was observed in CT + MFLX group (10%↑), whereas a decreasing trend persisted in GK + MFLX group (30%↓) (Supplementary Figure S2A). In normal rats, the unconjugated BAs levels were decreased significantly (30%↓) after MFLX treatment for 3 days, while conjugated BAs levels and the ratios of unconjugated to conjugated BAs (Uncon/Con ratio) were not changed. After MFLX treatment for 14 days, the unconjugated BAs levels were decreased significantly (54%↓) and Uncon/Con ratio were elevated (160%↑) (Supplementary Figure S1B, S2B). In addition, SBAs levels in CT + MFLX group were significantly reduced (95%↓) and the ratios of PBAs to SBAs (PBA/SBA ratio) were significantly increased (332%↑) after MFLX treatment for 3 days (Figure 2C; Supplementary Figure S1C), while no significant changes were observed in these BAs after 14 days (Figure 3C; Supplementary Figure S2C). After 3 days of MFLX treatment, In GK + MFLX group, levels of the unconjugated BAs (78%↓), conjugated BAs (48%↓), PBAs (76%↓), and SBAs (82%↓) were significantly reduced compared with GK group. Additionally, Uncon/Con ratio declined by 58%, while PBA/SBA ratio exhibited a modest increase (8%↑) (Supplementary Figure S1B, C; Figure 2C). After 14 days of treatment, there were no significant changes in these metrics, except for a notable increase in PBA/SBA ratio (40%↑) (Supplementary Figures S2B, C; Figure 3C).

In fecal samples, total BAs levels also exhibited a decreasing trend following MFLX treatment for 3 days (normal rats 73%↓; GK rats 57%↓) (Supplementary Figure S1D) and 14 days (normal rats 43%↓; GK rats 61%↓) (Supplementary Figure S2D). In normal rats, levels of conjugated BAs decreased by 76% after 3 days and by 45% after 14 days, while unconjugated BAs showed a similar reduction (3 days 73%↓; 14 days 43%↓). However, no significant changes were observed in Uncon/Con ratio (Supplementary Figure S1E, S2E). At the same time, PBAs increased (3 days 304%↑; 14 days 67%↑) and SBAs decreased (3 days 93%↓; 14 days 44%↓), resulting in an increase in PBA/SBA ratio (3 days 95%↑; 14 days 190%↑) (Figures 2D, 3D; Supplementary Figure S1F, S2F). In GK + MFLX group, MFLX treatment significantly reduced levels of the unconjugated BAs (3 days 57%↓; 14 days 61%↓) compared with GK group, whereas conjugated BAs remained relatively unchanged. The Uncon/Con ratio was significantly reduced (3 days 48%↓; 14 days 49%↓) (Supplementary Figure S1E, S2E). Furthermore, PBAs were significantly elevated (3 days 149%↑; 14 days 130%↑), while SBAs were substantially decreased (3 days 98%↓; 14 days 56%↓) (Figures 2D, 3D), resulting in a significant increase in the PBA/SBA ratio (3 days 154%↑; 14 days 95%↑) (Supplementary Figure S1F, S2F).

### 3.3 Alterations in intestinal SBAs levels and the effects on GLP-1 secretion *in vitro*

We conducted an analysis of intestinal BAs using a linear regression model incorporating interaction terms. The results revealed that MFLX had the most significant intervention effects on the PBAs CA (Drug_ES = 2.43) and CDCA (Drug_ES = 3.49), as well as the SBAs DCA (Drug_ES = −6.78), LCA (Drug_ES = −7.95) after treatment for 3 days. Among these, DCA was unique (Inter_ES = 3.21) in showing a drug effect modified by disease status, indicating that DCA, inherently lower in diabetic status, was further reduced by MFLX intervention (Supplementary Figure S3A). However, after 14 days treatment, the intervention effect of MFLX on these BAs was reduced [DCA (Drug_ES = −3.35), LCA (Drug_ES = −2.71)] (Supplementary Figure S3B).

**FIGURE 4:**
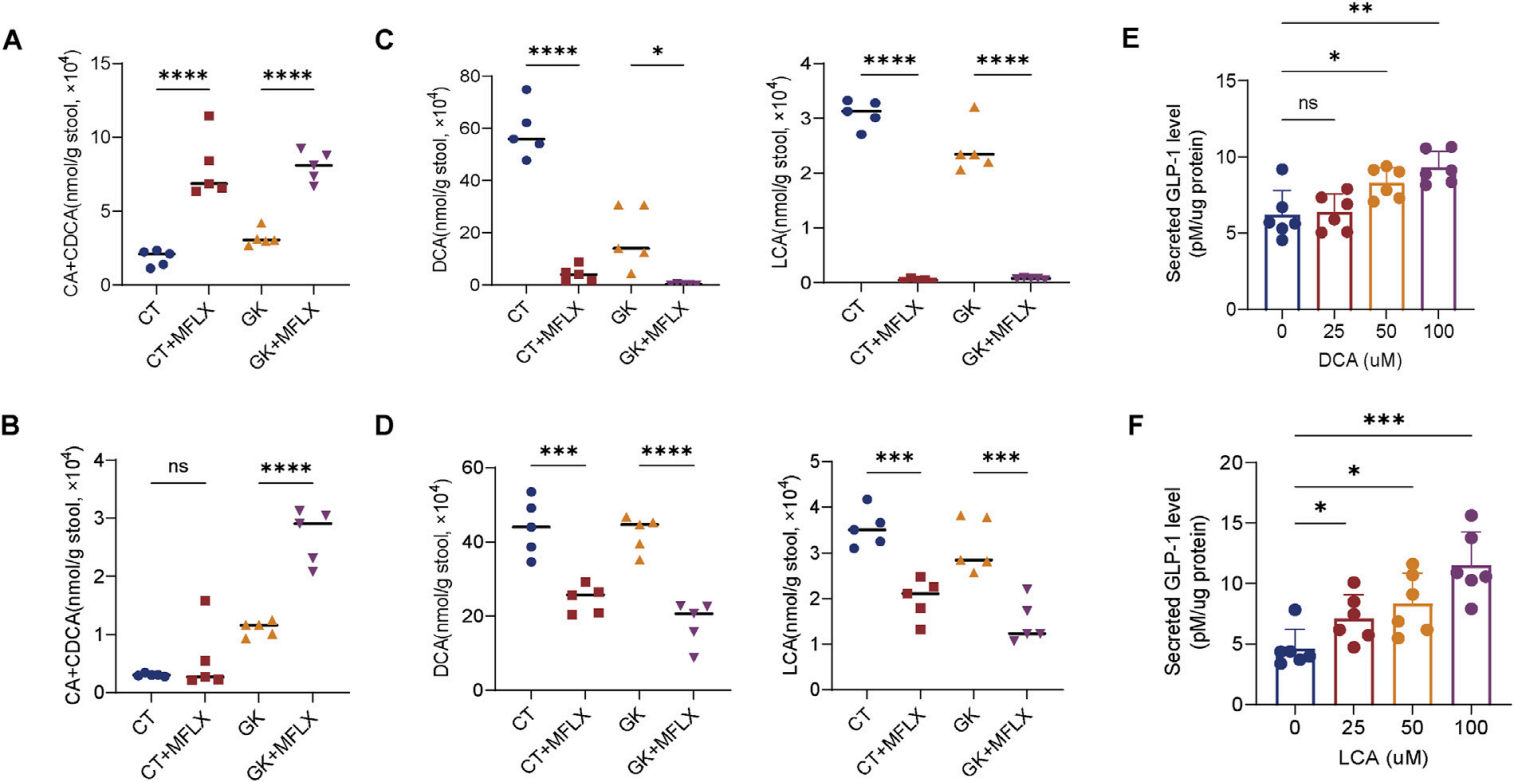
Alterations in intestinal unconjugated PBAs and SBAs levels. **(A, B)** Levels of CA and CDCA, DCA and LCA in feces after 3 days **(A)** and 14 days **(B)** of MFLX treatment; **(C, D)** Concentrations of DCA and LCA in feces after 3 days **(C)** and 14 days **(D)** of MFLX administration; **(E, F)** Levels of GLP-1 after 2-h incubation with DCA **(E)** and LCA **(F)** in NCI-H716 cells. All p-values were calculated using a two-tailed Student’s t-test, *P < 0.05, **P < 0.01, ***P < 0.001, ****P < 0.0001. Data are presented as the mean ± SD.

The levels of unconjugated PBAs CA + CDCA were significantly elevated in GK + MFLX group compared with GK group (3 days 151%↑; 14 days 144%↑) (Figures 4A, B), while unconjugated SBAs DCA and LCA were significantly reduced (3 days 98%↓; 14 days 57%↓) (Figures 4C, D), indicating that MFLX intervention inhibited the conversion of intestinal PBAs to SBAs. We found that the concentration of SBAs in the intestine was higher than that of PBAs. Since SBAs are stronger agonists of TGR5 compared to PBAs, and they significantly stimulated the secretion of intestinal GLP-1 (Castellanos-Jankiewicz et al., 2021; Gao et al., 2022; Jiao et al., 2022). We further confirmed that DCA and LCA promoted GLP-1 secretion in a concentration-dependently manner in NCI-H716 cells (Figures 4E, F). These results suggested that the inhibition of MFLX on intestinal SBAs led to a decrease in GLP-1 secretion, which was an important mechanism for MFLX-induced hyperglycemia.

### 3.4 MFLX-induced changes in gut microbiota and association between gut microbiota abundance and BAs profiles

Given that antibiotics primarily alter BAs composition and concentration by modifying the gut microbiota, we performed 16S rRNA sequencing on fecal samples from rats to evaluate the effect of MFLX on gut microbiota. Alpha diversity analysis revealed that Chao indices were significantly reduced in both normal and GK rats after MFLX treatment (Supplementary Figures S4A, B). Beta diversity indicated that the composition and abundance of the microbiota were more profoundly altered in GK rats compared to in normal rats after MFLX treatment (Supplementary Figure S4C, D).

**FIGURE 5:**
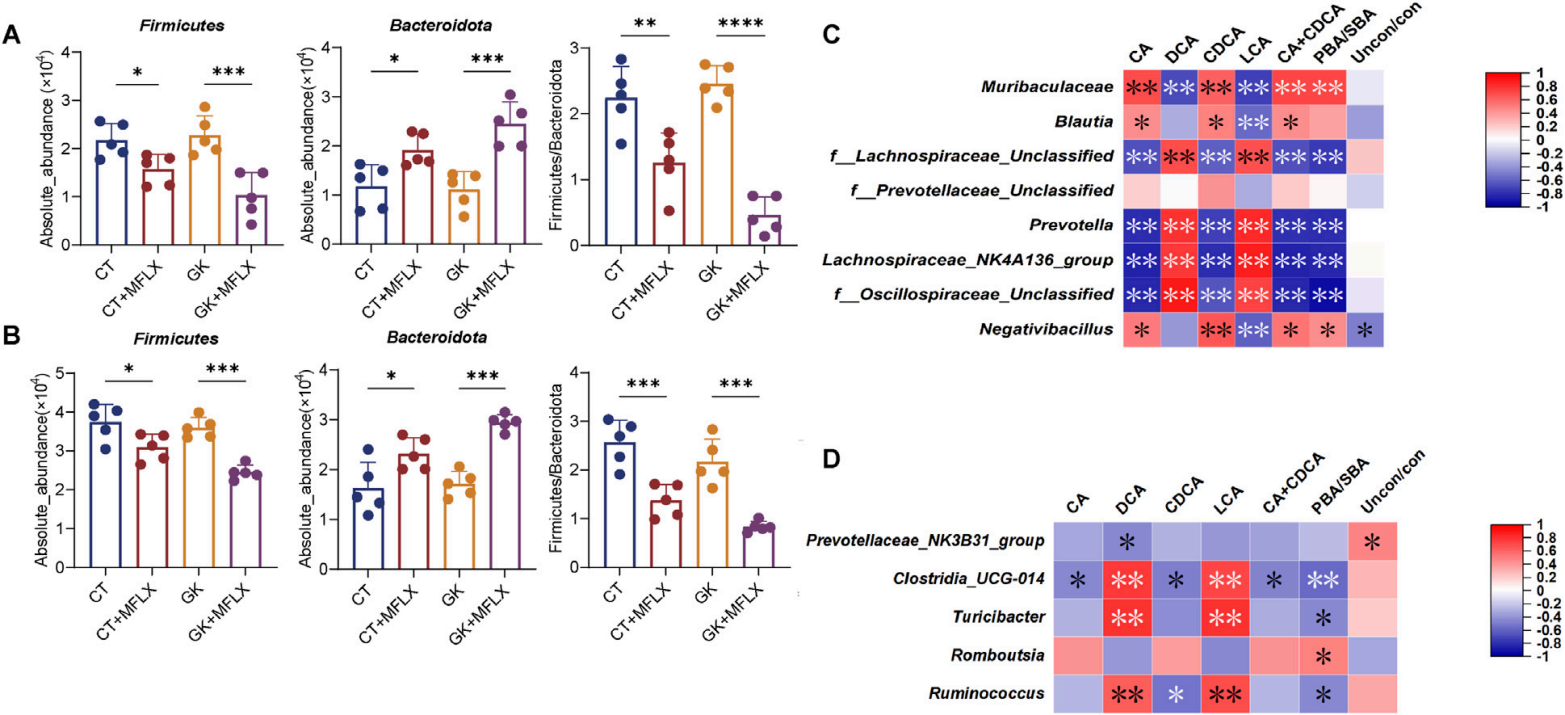
MFLX-induced changes in gut microbiota. **(A, B)** Absolute abundance of the phylum Firmicute and Bacteroidetes in the intestine after 3 days **(A)** and 14 days **(B)** of MFLX treatment; **(C, D)** Correlation between fecal SBAs levels and bacterial genera identified from linear regression analysis. Legends indicate relative abundances and correlation values. Respectively. Data are presented as the mean ± SD, *P < 0.05, **P < 0.01, ***P < 0.001, ****P < 0.0001.

At the phylum level, we observed a significant reduction in the abundance of the *Firmicutes* (normal rats: 3 days 28%↓,14 days 18% ↓; GK rats: 3 days 55%↓, 14 days 32%↓) and a notable increase in *Bacteroidetes* abundance (normal rats: 3 days 63%↑,14 days 43% ↑; GK rats: 3 days 120%↑, 14 days 72%↑) after MFLX treatment, resulting in a significantly reduced *Firmicutes/Bacteroidetes* ratio (Figures 5A, B).

At the genus level, we generated a heatmap of the relative abundance of the top 31 genera, which included 25 genera from *Firmicutes* and 6 from *Bacteroidetes* (Supplementary Figure S4E, F). Further analysis of these genera using a linear regression model with interaction terms revealed that after 3 days of MFLX treatment (Supplementary Figure S4G), the genera most affected were *Prevotella* (Drug_ES = −6.05), *Negativibacillus* (Drug_ES = 5.19), *f_Oscillospiraceae_Unclassified* (Drug_ES = −4.89), *f_Prevotellaceae_Unclassified* (Drug_ES = −3.72), *f_Lachnospiraceae_Unclassified* (Drug_ES = 3.3), *Blautia* (Drug_ES = 3.25), and *Lachnospiraceae_NK4A136_group* (Drug_ES = −3.04). After 14 days of treatment (Supplementary Figure S1H), the most affected genera were *f_Prevotellaceae_Unclassified* (Drug_ES = 4.51), *Romboutsia* (Drug_ES = 2.5), *Ruminococcus* (Drug_ES = −2.34), *Turicibacter* (Drug_ES = 2.26), *Clostridia_UCG-004* (Drug_ES = −2.17).

We further analyzed the relationship between BAs and the genera identified from linear regression analysis. Spearman’s correlation analysis indicated that after 3 days of MFLX treatment (Figure 5C), reduced levels of DCA and LCA levels were significantly and positively correlated with three members of the phylum *Firmicutes (f_Oscillospiraceae_Unclassified*, *f_Lachnospiraceae_Unclassified*, and *Lachnospiraceae_NK4A136_group)* and one member of the phylum *Bacteroidetes* (*Prevotella*). After 14 days of MFLX treatment (Figure 5D), DCA and LCA levels were significantly and positively correlated with *Ruminococcus*, *Turicibacter* and *Clostridia_UCG-004*, all of which belonged to the phylum *Firmicutes*.

### 3.5 Effect of MFLX on ileum FXR and TGR5

**FIGURE 6:**
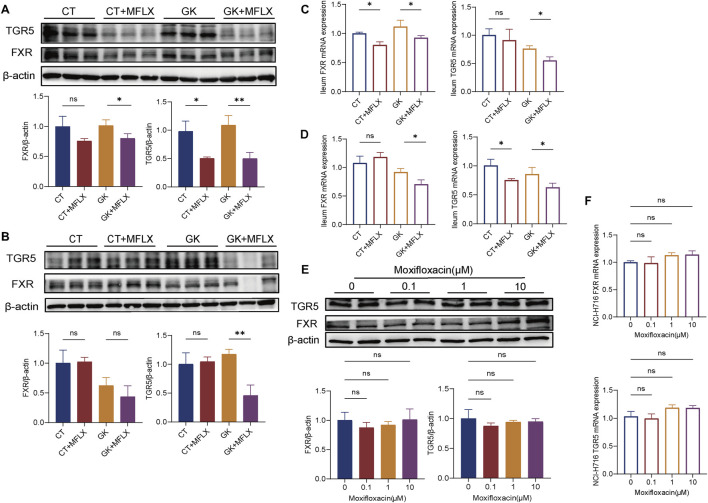
Effect of MFLX on ileum FXR and TGR5. **(A, B)** Relative expression of TGR5 and FXR proteins in the ileum of rats after 3 days **(A)** and 14 days **(B)** of MFLX treatment; **(C, D)** Relative expression of TGR5 and FXR mRNAs in the ileum of rats after 3 days **(C)** and 14 days **(D)** of MFLX treatment; **(E, F)** Relative expression of TGR5 and FXR proteins **(E)** and mRNAs **(F)** in NCI-H716 cells following 24 h of MFLX treatment.

Since we observed decreased DCA and LCA levels in the gut, we further examined the expression levels of pivotal BAs receptors TGR5 and FXR. After 3 days (Figures 6A, C) and 14 days (Figures 6B, D) of MFLX treatment, both protein and mRNA expression of the intestinal BAs receptors TGR5 and FXR showed a decreasing trend in CT + MFLX group compared with CT group, and the decrease was more obvious in GK + MFLX. Additionally, in NCI-H716 cells, MFLX administration was found to have no effect on TGR5 and FXR protein and mRNA expression (Figures 6E, F).

### 3.6 Effect of MFLX on hepatic metabolic enzymes

**FIGURE 7:**
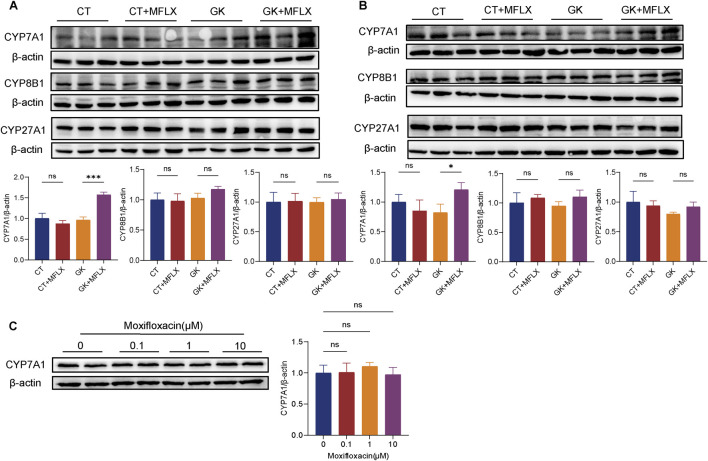
Effect of MFLX on hepatic metabolic enzymes. **(A, B)** Relative expression of CYP7A1, CYP8B1 and CYP27A1 in the liver of rats following 3 days **(A)** and 14 days **(B)** of MFLX treatment; **(C)** Relative expression of CYP7A1 proteins in HepG2 cells after 24 h of MFLX treatment.

Given the observed reduction intestinal FXR protein and mRNA, along with a significant decrease in serum FGF15 levels, the hepatic metabolizing enzyme CYP7A1, CYP8B1, and CYP27A1 protein were measured. Results indicated that CYP7A1 expression was elevated following MFLX treatment in GK rats (Figures 7A, B). There were no significant changes on expression of CYP8A1 and CYP27A1 in normal rats and GK rats after MFLX treatment. *In vitro*, MFLX did not exert a direct effect on CYP7A1 in HepG2 cells (Figure 7C).

## 4 Discussion

MFLX is widely used as a first-line anti-infective chemotherapeutic agent due to its favorable tolerability and broad-spectrum antimicrobial activity (Du et al., 2021; Wang H. et al., 2023). However, MFLX-induced hyperglycemia cannot be overlooked during the course of treatment. Therefore, a clear understanding of the mechanisms by how MFLX affects energy metabolism is essential to mitigate these adverse effects.

Growing evidence suggests that the crosstalk between BAs metabolism and the gut microbiota plays a key role in maintaining nutrient metabolism and energy balance (Li et al., 2022; Sun et al., 2018; Chen et al., 2023; Wahlström et al., 2016). In the present study, we investigated the short-term (3 days) and long-term (14 days) effects of MFLX on glucose metabolism in both normal and GK rats. Our findings revealed that MFLX induced hyperglycemia in diabetic rats, with a more pronounced reduction in serum insulin,GLP-1, and FGF15 levels than observed in normal rats. Concurrently, MFLX treatment led to alterations in the gut microbiota community that inhibited SBAs production. This suppression downregulated both protein and mRNA expression of intestinal TGR5, further reducing GLP-1 secretion, which was identified as a significant contributor to MFLX-induced hyperglycemia. Additionally, the reduction in total BAs levels, coupled with the inhibition of intestinal FXR, triggered a negative feedback mechanism, resulting in the upregulation of hepatic BAs metabolic enzyme.

The risk of dysglycemia is elevated in the diabetic state following fluoroquinolone use (Chou et al., 2013; Bartoletti et al., 2022; Saad et al., 2021), and our study confirmed that MFLX had a more pronounced impact on diabetic rats. Specifically, MFLX treatment significantly elevated blood glucose levels in GK rats, while no notable changes in blood glucose, insulin, GLP-1, or FGF15 levels were observed in normal rats. Furthermore, our study demonstrated that short-term MFLX treatment (3 days) had a more substantial effect on glucose metabolism in diabetic rats than long-term treatment (14 days). After 3 days of MFLX administration in GK rats, insulin, GLP-1, and FGF15 levels were markedly reduced. 14-day treatment of MFXL reduced FGF15 only.

In our study, both serum and fecal samples demonstrated a declining trend in total BAs levels in normal and GK rats after MFLX treatment. Intestinal SBAs levels were reduced in normal rats, and further decreased in GK rats after MFLX treatment. In GK rats, the linear model with interaction terms revealed that DCA and LCA levels in the gut exhibited the most significant reduction, with DCA decreasing by 98% after 3 days and 57% after 14 days, and LCA decreasing by 97% after 3 days and 53% after 14 days. It is well-established that DCA and LCA are metabolized from CA and CDCA through specific gut microbiota (Kiriyama and Nochi, 2023; Funabashi et al., 2020; Su et al., 2023). The 16S rRNA sequencing results showed that MFLX significantly reduced the species richness of normal and GK rats. Meanwhile, compared with normal rats, MFLX had a more significant effect on the composition and abundance of gut microbiota in GK rats. At the genus level, after 3 days of MFLX treatment, several genera *f_Lachnospiraceae_Unclassified*, *Lachnospiraceae_NK4A136_*group, and *Prevotella* were significantly and positively correlated with the reduced levels of DCA and LCA, and after 14 days of MFLX treatment, DCA and LCA levels were significantly and positively correlated with *Ruminococcus*, *Turicibacter* and *Clostridia_UCG-004*. Previous studies have showed that increased relative abundances of *Prevotella*, *Lachnospiraceae*, and *Ruminococcus* enhanced SBAs production (McMillan and Theriot, 2024; Lin et al., 2024; Ma et al., 2024; Zhou et al., 2023; Zhang et al., 2023). In this study, we found that the effect of short-term and long-term intervention of MFLX on intestinal bacteria was different, likely due to either the development of tolerance among gut microbiota to MFLX or the initiation of self-regulation mechanisms. This differential response may explain why changes in gut concentration of DCA and LCA were more significant after 3 days compared to 14 days.

TGR5 is a pivotal receptor involved in the regulation of BA-mediated energy homeostasis (Castellanos-Jankiewicz et al., 2021; Gao et al., 2022; Jiao et al., 2022; Lun et al., 2024). In this study, we observed that MFLX downregulated both protein and mRNA levels of intestinal TGR5, and subsequently led to decreased GLP-1 secretion and elevated blood glucose levels. It is well established that DCA and LCA are the most potent agonists of intestinal TGR5 (Gao et al., 2022; Jiao et al., 2022; Duboc et al., 2014). Numerous studies have identified BAs as robust triggers of GLP-1 release (Tian et al., 2022; Li et al., 2020; Zhai et al., 2018; Hui et al., 2020), with BAs-stimulated GLP-1 secretion significantly reduced in TGR5 knockout models (Selwyn et al., 2015; Harach et al., 2012; Brighton et al., 2015; Li et al., 2023). In our study, after MFLX intervention, the intestinal SBAs was significantly reduced, and it was further decreased in GK rats compared with normal rats. Therefore, we speculated that suppressed intestinal SBA production is a critical mechanism underlying MFLX-induced hyperglycemia.

It was also found that the expression of intestinal FXR was downregulated after moxifloxacin intervention, which was more obvious in GK rats. It has been reported that FXR activation inhibited GLP-1 secretion, which appears contradictory to the effects mediated by TGR5 activation (Zheng et al., 2021; Trabelsi et al., 2015); however, research conclusions on the regulatory effects of FXR on GLP-1 secretion are not consistent. Pathak et al. (2018) showed that intestine-restricted FXR agonist fexaramine shapes the gut microbiota to produce more LCA, and thus activates TGR5-GLP-1 signaling to improve metabolism. A recent study suggested that activation of TGR5, but not FXR, stimulated GLP-1 release *in vitro* and *in vivo* (Wang Q. et al., 2023). FXR signaling in the gut-liver axis plays an important role in maintaining glucose metabolism and BAs homeostasis. It was showed that activated hepatic FXR signaling pathway was related to promoted BA synthesis-related protein expressions, inhibited inflammation and gluconeogensis, as well as improved insulin resistance (Du et al., 2024). On the other hand, intestinal FXR activation promotes the transcription of FGF 15/19, a hormone with insulin-mimetic properties that inhibits hepatic gluconeogenesis (Sadowska et al., 2023; Jin et al., 2023; de Carvalho et al., 2024; Schaap, 2012). Intestinal bile acids as FXR ligands can directly regulate intestinal FXR expression, and also indirectly modulate hepatic FXR signaling through the enterohepatic circulation, thereby influencing glucose metabolism (Ticho et al., 2019). In our study, MFLX treatment was associated with a reduction of intestinal FXR expression, alongside decreased serum FGF15 concentrations. This downregulation in FXR-FGF15 signaling may constitute an additional mechanism contributing to MFLX-induced hyperglycemia. Recent studies have demonstrated that gut microbiota-mediated production of SBAs modulates hepatic *de novo* BAs synthesis through the FXR-FGF15 axis in mice (Wu et al., 2023; Liu et al., 2021; Cao et al., 2017). In our study, MFLX treatment resulted in downregulation of intestinal FXR and a reduction in serum FGF15 levels, accompanied by an observed trend of hepatic CYP7A1 upregulation in GK rats. Given that MFLX significantly reduced BAs concentrations *in vivo*, the inhibition of FXR likely triggered a compensatory feedback mechanism, thereby increasing the expression of CYP7A1, the primary hepatic enzyme responsible for BAs metabolism.

In the present study, we investigated the critical role of the gut microbiota-derived BAs in MFLX-induced hyperglycemia, potentially through decreased intestinal TGR5 pathway. Additionally, our findings suggested that FXR-FGF15 axis may play a contributory role modulating hepatic BAs synthesis during MFLX treatment. These results offer insights into the underlying mechanisms of MFLX-induced hyperglycemia and provide a foundational basis for guiding the clinical use of MFLX to minimize metabolic side effects.

## 5 Conclusion

**FIGURE 8:**
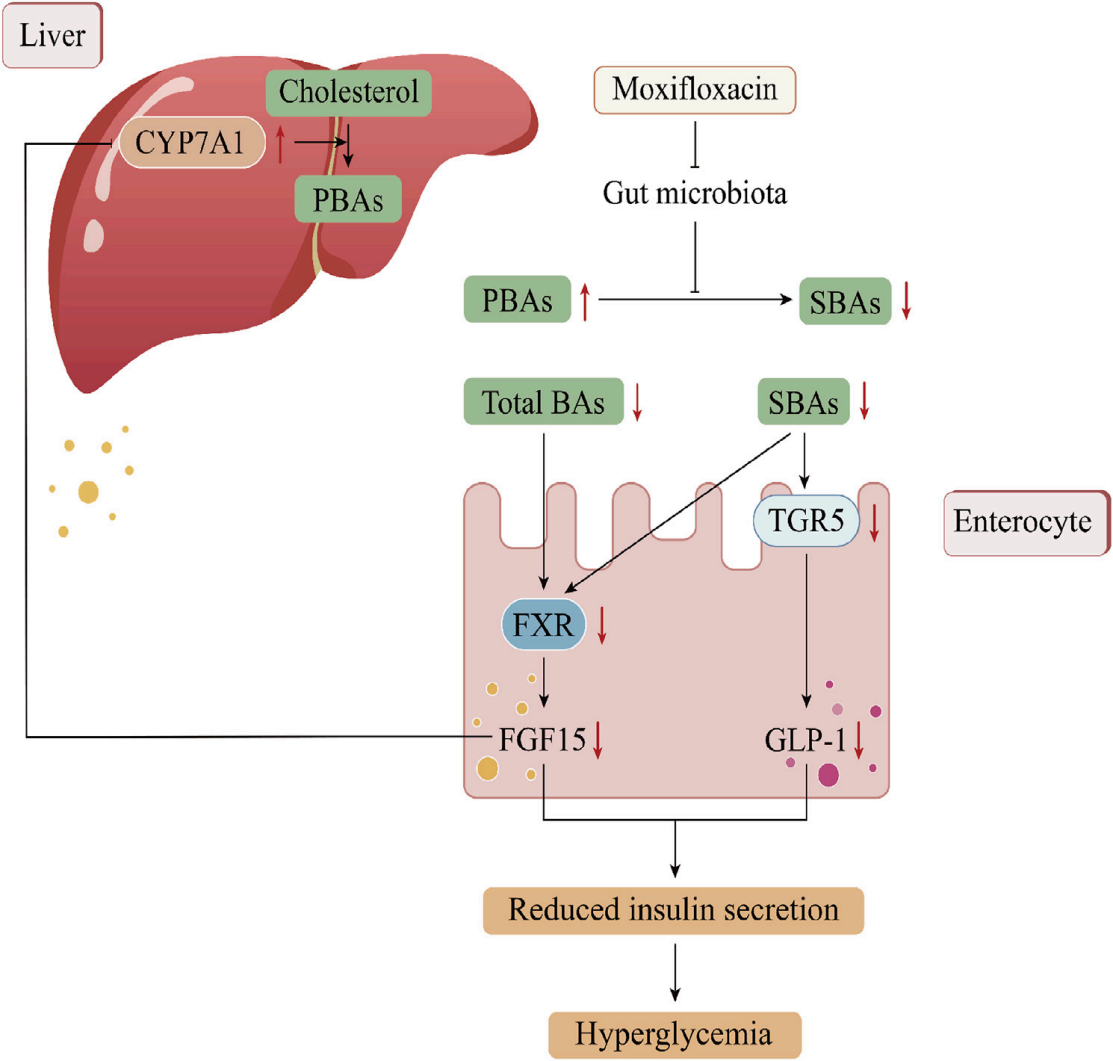
The mechanism of moxifloxacin-induced hyperglycemia in this study.

To the best of our knowledge, the use of fluoroquinolone antibiotics has been linked to dysglycemia, with clinical cases documenting cases of MFLX-induced glucose dysregulation. Here, we found that MFLX posed a risk of further exacerbation of blood glucose levels in diabetic rats and investigated the possible mechanisms involving the gut microbiota-SBAs-TGR5/FXR pathway (Figure 8). Our findings provide a scientific basis for the early identification and management of dysglycemia risk associated with clinical use of fluoroquinolone antibiotics.

## Data Availability

The data presented in the study are deposited in the SRA database of National Center for Biotechnology Information (NCBI) repository, BioProject accession number PRJNA1240372.

## References

[B1] Akash M. S. Rehman K. Chen S. (2013). Goto-Kakizaki rats: its suitability as non-obese diabetic animal model for spontaneous type 2 diabetes mellitus. Curr. Diabetes Rev. 9, 387–396. 10.2174/15733998113099990069 23855509

[B2] Althaqafi A. Ali M. Alzahrani Y. Ming L. C. Hussain Z. (2021). How safe are fluoroquinolones for diabetic patients? A systematic review of dysglycemic and neuropathic effects of fluoroquinolones. Ther. Clin. Risk Manag. 17, 1083–1090. 10.2147/Tcrm.S284171 34675522 PMC8520959

[B3] Bartoletti R. Claps F. Tulone G. Perotti A. Zucchi A. Riccardi N. (2022). Antibiotic prophylaxis in patients who had undergone to prostate biopsy in between the EMA warning era: effects of fluoroquinolones in diabetic and non-diabetic patients. Results of an observational cohort study. World J. Urology 40, 2025–2031. 10.1007/s00345-022-04055-7 PMC9279202 35689105

[B4] Beekman C. N. Penumutchu S. Peterson R. Han G. G. Belenky M. Hasan M. H. (2024). Spatial analysis of murine microbiota and bile acid metabolism during amoxicillin treatment. Cell. Rep. 43, 114572. 10.1016/j.celrep.2024.114572 39116202 PMC12232608

[B5] Behr C. Slopianka M. Haake V. Strauss V. Sperber S. Kamp H. (2019). Analysis of metabolome changes in the bile acid pool in feces and plasma of antibiotic-treated rats. Toxicol. Appl. Pharmacol. 363, 79–87. 10.1016/j.taap.2018.11.012 30502395

[B6] Belforti R. K. Lagu T. Haessler S. Lindenauer P. K. Pekow P. S. Priya A. (2016). Association between initial route of fluoroquinolone administration and outcomes in patients hospitalized for community-acquired pneumonia. Clin. Infect. Dis. 63, 1–9. 10.1093/cid/ciw209 27048748 PMC4901867

[B7] Brighton C. A. Rievaj J. Kuhre R. E. Glass L. L. Schoonjans K. Holst J. J. (2015). Bile acids trigger GLP-1 release predominantly by accessing basolaterally located G protein-coupled bile acid receptors. Endocrinology 156, 3961–3970. 10.1210/en.2015-1321 26280129 PMC4606749

[B8] Cao L. Che Y. Meng T. Deng S. Zhang J. Zhao M. (2017). Repression of intestinal transporters and FXR-FGF15 signaling explains bile acids dysregulation in experimental colitis-associated colon cancer. Oncotarget 8, 63665–63679. 10.18632/oncotarget.18885 28969019 PMC5609951

[B9] Cariello M. Piccinin E. Garcia-Irigoyen O. Sabbà C. Moschetta A. (2018). Nuclear receptor FXR, bile acids and liver damage: introducing the progressive familial intrahepatic cholestasis with FXR mutations. Biochimica Biophysica Acta-Molecular Basis Dis. 1864, 1308–1318. 10.1016/j.bbadis.2017.09.019 28965883

[B10] Castellanos-Jankiewicz A. Guzmán-Quevedo O. Fénelon V. S. Zizzari P. Quarta C. Bellocchio L. (2021). Hypothalamic bile acid-TGR5 signaling protects from obesity. Cell. Metab. 33, 1483-+. 10.1016/j.cmet.2021.04.009 33887197

[B11] Chen B. Bai Y. Tong F. Yan J. Zhang R. Zhong Y. (2023). Glycoursodeoxycholic acid regulates bile acids level and alters gut microbiota and glycolipid metabolism to attenuate diabetes. Gut Microbes 15, 2192155. 10.1080/19490976.2023.2192155 36967529 PMC10054359

[B12] Chiang J. Y. L. (2017). Bile acid metabolism and signaling in liver disease and therapy. Liver Res. 1, 3–9. 10.1016/j.livres.2017.05.001 29104811 PMC5663306

[B13] Chou H. W. Wang J. L. Chang C. H. Lee J. J. Shau W. Y. Lai M. S. (2013). Risk of severe dysglycemia among diabetic patients receiving levofloxacin, ciprofloxacin, or moxifloxacin in taiwan. Clin. Infect. Dis. 57, 971–980. 10.1093/cid/cit439 23948133

[B14] De Carvalho M. B. Jorge G. M. C. P. Zanardo L. W. Hamada L. M. Izabel L. D. Santoro S. (2024). The role of FGF19 in metabolic regulation: insights from preclinical models to clinical trials. Am. J. Physiology-Endocrinology Metabolism 327, E279–E289. 10.1152/ajpendo.00156.2024 39017679

[B15] Du H. Cui L. Zhao X. Yu Z. He T. Zhang B. (2024). Butylparaben induces glycolipid metabolic disorders in mice via disruption of gut microbiota and FXR signaling. J. Hazard Mater 474, 134821. 10.1016/j.jhazmat.2024.134821 38850927

[B16] Du X. W. Han Y. Jian Y. F. Chen L. P. Xuan J. W. (2021). Clinical benefits and cost-effectiveness of moxifloxacin as initial treatment for community-acquired pneumonia: a meta-analysis and economic evaluation. Clin. Ther. 43, 1894–1909.e1. 10.1016/j.clinthera.2021.03.006 33814200

[B17] Duboc H. Tache Y. Hofmann A. F. (2014). The bile acid TGR5 membrane receptor: from basic research to clinical application. Dig. Liver Dis. 46, 302–312. 10.1016/j.dld.2013.10.021 24411485 PMC5953190

[B18] Eckmann C. Tulkens P. M. (2021). Current and future options for treating complicated skin and soft tissue infections: focus on fluoroquinolones and long-acting lipoglycopeptide antibiotics. J. Antimicrob. Chemother. 76, iv9–iv22. 10.1093/jac/dkab351 34849999 PMC8632788

[B19] El Ghandour S. Azar S. T. (2015). Dysglycemia associated with quinolones. Prim. Care Diabetes 9, 168–171. 10.1016/j.pcd.2014.10.006 25466161

[B20] Fiorucci S. Biagioli M. Baldoni M. Ricci P. Sepe V. Zampella A. (2021). The identification of farcesoid X receptor modulators as treatment options for nonalcoholic fatty liver disease. Expert Opin. Drug Discov. 16, 1193–1208. 10.1080/17460441.2021.1916465 33849361

[B21] Funabashi M. Grove T. L. Wang M. Varma Y. McFadden M. E. Brown L. C. (2020). A metabolic pathway for bile acid dehydroxylation by the gut microbiome. Nature *.* 582, 566–570. 10.1038/s41586-020-2396-4 32555455 PMC7319900

[B22] Gao R. L. Meng X. J. Xue Y. L. Mao M. Liu Y. R. Tian X. W. (2022). Bile acids-gut microbiota crosstalk contributes to the improvement of type 2 diabetes mellitus. Front. Pharmacol. 13, 1027212. 10.3389/fphar.2022.1027212 36386219 PMC9640995

[B23] Gonzalez F. J. Jiang C. T. Xie C. Pattèrson A. D. (2017). Intestinal farnesoid X receptor signaling modulates metabolic disease. Dig. Dis. 35, 178–184. 10.1159/000450908 28249275 PMC6595218

[B24] Granados J. Ceballos M. Amariles P. (2018). Dysglycemia asociada a fluoroquinolones: una revisión estructurada. Rev. Med. Chil. 146, 618–626. 10.4067/s0034-98872018000500618 30148925

[B25] Han S. Y. Wang K. C. Shen J. Xia H. Lu Y. M. Zhuge A. X. (2023). Probiotic *Pediococcus pentosaceus* Li05 improves cholestasis through the FXR-SHP and FXR-FGF15 pathways Li05 improves cholestasis through the FXR-SHP and FXR-FGF15 pathways. Nutrients 15, 4864. 10.3390/nu15234864 38068723 PMC10708340

[B26] Harach T. Pols T. W. Nomura M. Maida A. Watanabe M. Auwerx J. (2012). TGR5 potentiates GLP-1 secretion in response to anionic exchange resins. Sci. Rep. 2, 430. 10.1038/srep00430 22666533 PMC3362799

[B27] Hui S. C. Huang L. Wang X. L. Zhu X. H. Zhou M. Chen M. T. (2020). Capsaicin improves glucose homeostasis by enhancing glucagon-like peptide-1 secretion through the regulation of bile acid metabolism via the remodeling of the gut microbiota in male mice. Faseb J. 34, 8558–8573. 10.1096/fj.201902618RR 32359008

[B28] Iguchi T. Goto K. Watanabe K. Hashimoto K. Suzuki T. Kishino H. (2020). Fluoroquinolones suppress gluconeogenesis by inhibiting fructose 1,6-bisphosphatase in primary monkey hepatocytes. Toxicol. Vitro 65, 104786. 10.1016/j.tiv.2020.104786 32004540

[B29] Ishiwata Y. Takahashi Y. Nagata M. Yasuhara M. (2013). Effects of moxifloxacin on serum glucose concentrations in rats. Biol. Pharm. Bull. 36, 686–690. 10.1248/bpb.b12-00930 23358329

[B30] Jia W. Xie G. X. Jia W. P. (2018). Bile acid-microbiota crosstalk in gastrointestinal inflammation and carcinogenesis. Nat. Rev. Gastroenterology and Hepatology 15, 111–128. 10.1038/nrgastro.2017.119 29018272 PMC5899973

[B31] Jiao T. Y. Ma Y. D. Guo X. Z. Ye Y. F. Xie C. (2022). Bile acid and receptors: biology and drug discovery for nonalcoholic fatty liver disease. Acta Pharmacol. Sin. 43, 1103–1119. 10.1038/s41401-022-00880-z 35217817 PMC9061718

[B32] Jin L. G. Yang R. Y. Geng L. L. Xu A. M. (2023). Fibroblast growth factor-based pharmacotherapies for the treatment of obesity-related metabolic complications. Annu. Rev. Pharmacol. Toxicol. 63, 359–382. 10.1146/annurev-pharmtox-032322-093904 36100222

[B33] Juhl C. R. Burgdorf J. Knudsen C. Lubberding A. F. Veedfald S. Isaksen J. L. (2023). A randomized, double-blind, crossover study of the effect of the fluoroquinolone moxifloxacin on glucose levels and insulin sensitivity in young men and women. Diabetes Obes. Metab. 25, 98–109. 10.1111/dom.14851 36054143 PMC10087839

[B34] Kabbara W. K. Ramadan W. H. Rahbany P. Al-Natour S. (2015). Evaluation of the appropriate use of commonly prescribed fluoroquinolones and the risk of dysglycemia. Ther. Clin. Risk Manag. 11, 639–647. 10.2147/TCRM.S81280 25960658 PMC4410896

[B35] Katafuchi T. Makishima M. (2022). Molecular basis of bile acid-FXR-FGF15/19 signaling Axis. Int. J. Mol. Sci. 23, 6046. 10.3390/ijms23116046 35682726 PMC9181207

[B36] Kim K. Park M. Lee Y. M. Rhyu M. R. Kim H. Y. (2014). Ginsenoside metabolite compound K stimulates glucagon-like peptide-1 secretion in NCI-H716 cells via bile acid receptor activation. Archives Pharmacal Res. 37, 1193–1200. 10.1007/s12272-014-0362-0 24590628

[B37] Kiriyama Y. Nochi H. (2023). The role of gut microbiota-derived lithocholic acid, deoxycholic acid and their derivatives on the function and differentiation of immune cells. Microorganisms 11, 2730. 10.3390/microorganisms11112730 38004742 PMC10672800

[B38] Li M. Wang S. Li Y. Zhao M. Kuang J. Liang D. (2022). Gut microbiota-bile acid crosstalk contributes to the rebound weight gain after calorie restriction in mice. Nat. Commun. 13, 2060. 10.1038/s41467-022-29589-7 35440584 PMC9018700

[B39] Li M. Zhou W. Dang Y. Li C. Ji G. Zhang L. (2020). Berberine compounds improves hyperglycemia via microbiome mediated colonic TGR5-GLP pathway in db/db mice. Biomed. Pharmacother. 132, 110953. 10.1016/j.biopha.2020.110953 33254441

[B40] Li W. Zhuang T. Wang Z. Wang X. Liu L. Luo Y. (2023). Red ginseng extracts ameliorate high-fat diet-induced obesity and insulin resistance by activating the intestinal TGR5-mediated bile acids signaling pathway. Phytomedicine 119, 154982. 10.1016/j.phymed.2023.154982 37531904

[B41] Lin J. Liu H. Sun Y. Zou J. Nie Q. Nie S. (2024). Arabinoxylan alleviates obesity by regulating gut microbiota and bile acid metabolism. J. Agric. Food Chem. 72, 23295–23305. 10.1021/acs.jafc.4c06392 39400044

[B42] Liu L. Yang M. Dong W. Liu T. Song X. Gu Y. (2021). Gut dysbiosis and abnormal bile acid metabolism in colitis-associated cancer. Gastroenterol. Res. Pract. 2021, 6645970. 10.1155/2021/6645970 33708251 PMC7929689

[B43] Lun W. J. Yan Q. H. Guo X. H. Zhou M. C. Bai Y. He J. C. (2024). Mechanism of action of the bile acid receptor TGR5 in obesity. Acta Pharm. Sin. B 14, 468–491. 10.1016/j.apsb.2023.11.011 38322325 PMC10840437

[B44] Ma X. Qiu Y. Mao M. Lu B. Zhao H. Pang Z. (2024). PuRenDan alleviates type 2 diabetes mellitus symptoms by modulating the gut microbiota and its metabolites. J. Ethnopharmacol. 322, 117627. 10.1016/j.jep.2023.117627 38147943

[B45] McMillan A. S. Theriot C. M. (2024). Bile acids impact the microbiota, host, and *C. difficile* dynamics providing insight into mechanisms of efficacy of FMTs and microbiota-focused therapeutics. Gut Microbes 16, 2393766. 10.1080/19490976.2024.2393766 39224076 PMC11376424

[B46] Münzker J. Haase N. Till A. Sucher R. Haange S. B. Nemetschke L. (2022). Functional changes of the gastric bypass microbiota reactivate thermogenic adipose tissue and systemic glucose control via intestinal FXR-TGR5 crosstalk in diet-induced obesity. Microbiome 10, 96. 10.1186/s40168-022-01264-5 35739571 PMC9229785

[B47] Pathak P. Xie C. Nichols R. G. Ferrell J. M. Boehme S. Krausz K. W. (2018). Intestine farcesoid X receptor agonist and the gut microbiota activate G-protein bile acid receptor-1 signaling to improve metabolism. Hepatology 68, 1574–1588. 10.1002/hep.29857 29486523 PMC6111007

[B48] Perino A. Velázquez-Villegas L. A. Bresciani N. Sun Y. Huang Q. Y. Fénelon V. S. (2021). Central anorexigenic actions of bile acids are mediated by TGR5. Nat. Metab. 3, 595–603. 10.1038/s42255-021-00398-4 34031591 PMC7610881

[B49] Qi Y. C. Shi L. K. Duan G. Z. Ma Y. G. Li P. F. (2021). Taurochenodeoxycholic acid increases cAMP content via specially interacting with bile acid receptor TGR5. Molecules 26, 7066. 10.3390/molecules26237066 34885648 PMC8659238

[B50] Qiu H. Y. Yuan S. S. Yang F. Y. Shi T. T. Yang J. K. (2016). HERG protein plays a role in moxifloxacin-induced hypoglycemia. J. Diabetes Res. 2016, 6741745. 10.1155/2016/6741745 26649323 PMC4663361

[B51] Saad N. A. Elberry A. A. Samy Matar H. Hussein R. R. S. (2021). Effect of ciprofloxacin vs levofloxacin on QTc-interval and dysglycemia in diabetic and non-diabetic patients. Int. J. Clin. Pract. 75, e14072. 10.1111/ijcp.14072 33559294

[B52] Sadowska A. Poniedzialek-Czajkowska E. Mierzynski R. Alonso-Magdalena P. (2023). The role of the FGF19 family in the pathogenesis of gestational diabetes: a narrative review. Int. J. Mol. Sci. 24, 17298. 10.3390/ijms242417298 38139126 PMC10743406

[B53] Schaap F. G. (2012). Role of fibroblast growth factor 19 in the control of glucose homeostasis. Curr. Opin. Clin. Nutr. Metab. Care 15, 386–391. 10.1097/MCO.0b013e3283547171 22617565

[B54] Selwyn F. P. Csanaky I. L. Zhang Y. C. Klaassen C. D. (2015). Importance of large intestine in regulating bile acids and glucagon-like peptide-1 in germ-free mice. Drug Metabolism Dispos. 43, 1544–1556. 10.1124/dmd.115.065276 PMC4576674 26199423

[B55] Singh P. Walia V. Verma P. K. (2023). Hypoglycemia and anxiolysis mediated by levofloxacin treatment in diabetic rats. J. Diabetes Metab. Disord. 22, 1197–1209. 10.1007/s40200-023-01234-0 37975146 PMC10638278

[B56] Su X. M. Gao Y. H. Yang R. C. (2023). Gut microbiota derived bile acid metabolites maintain the homeostasis of gut and systemic immunity. Front. Immunol. 14, 1127743. 10.3389/fimmu.2023.1127743 37256134 PMC10225537

[B57] Sun L. Xie C. Wang G. Wu Y. Wu Q. Wang X. (2018). Gut microbiota and intestinal FXR mediate the clinical benefits of metformin. Nat. Med. 24, 1919–1929. 10.1038/s41591-018-0222-4 30397356 PMC6479226

[B58] Ten Doesschate T. Kuiper S. van Nieuwkoop C. Hassing R. J. Ketels T. van Mens S. P. (2022). Fosfomycin vs ciprofloxacin as oral step-down treatment for *Escherichia coli* febrile urinary tract infections in women: a randomized, placebo-controlled, double-blind, multicenter trial. Clin. Infect. Dis. 75, 221–229. 10.1093/cid/ciab934 34791074 PMC8689999

[B59] Tian F. Y. Huang S. Xu W. D. Chen L. Su J. M. Ni H. X. (2022). Compound K attenuates hyperglycemia by enhancing glucagon-like peptide-1 secretion through activating TGR5 via the remodeling of gut microbiota and bile acid metabolism. J. Ginseng Res. 46, 780–789. 10.1016/j.jgr.2022.03.006 36312739 PMC9597441

[B60] Ticho A. L. Malhotra P. Dudeja P. K. Gill R. K. Alrefai W. A. (2019). Intestinal absorption of bile acids in Health and disease. Compr. Physiol. 10, 21–56. 10.1002/cphy.c190007 31853951 PMC7171925

[B61] Trabelsi M. S. Daoudi M. Prawitt J. Ducastel S. Touche V. Sayin S. I. (2015). Farnesoid X receptor inhibits glucagon-like peptide-1 production by enteroendocrine L cells. Nat. Commun. 6, 7629. 10.1038/ncomms8629 26134028 PMC4579574

[B62] Wahlström A. Sayin S. I. Marschall H. U. Bäckhed F. (2016). Intestinal crosstalk between bile acids and microbiota and its impact on host metabolism. Cell. Metab. 24, 41–50. 10.1016/j.cmet.2016.05.005 27320064

[B63] Wang H. Liu H. Lou M. Xu L. Zhang W. Jing L. (2023a). Comprehensive clinical evaluation of moxifloxacin: a retrospective study. Med. Baltim. 102, e33896. 10.1097/MD.0000000000033896 PMC10238022 37266643

[B64] Wang Q. Lin H. Shen C. Zhang M. Wang X. Yuan M. (2023b). Gut microbiota regulates postprandial GLP-1 response via ileal bile acid-TGR5 signaling. Gut Microbes 15, 2274124. 10.1080/19490976.2023.2274124 37942583 PMC10730136

[B65] Wu W. Kaicen W. Bian X. Yang L. Ding S. Li Y. (2023). Akkermansia muciniphila alleviates high-fat-diet-related metabolic-associated fatty liver disease by modulating gut microbiota and bile acids. Microb. Biotechnol. 16, 1924–1939. 10.1111/1751-7915.14293 37377410 PMC10527187

[B66] Ye X. Huang D. Dong Z. X. Wang X. X. Ning M. Xia J. (2023). FXR signaling-mediated bile acid metabolism is critical for alleviation of cholesterol gallstones by lactobacillus strains. Microbiol. Spectr. 11, e00518–e00522. 10.1128/spectrum.05072-22 PMC9603329 36036629

[B67] Zeng Y. Wu Y. F. Zhang Q. Xiao X. H. (2024). Crosstalk between glucagon-like peptide 1 and gut microbiota in metabolic diseases. Mbio 15, e0203223. 10.1128/mbio.02032-23 38055342 PMC10790698

[B68] Zhai H. Li Z. Peng M. Huang Z. Qin T. Chen L. (2018). Takeda G protein-coupled receptor 5-mechanistic target of rapamycin complex 1 signaling contributes to the increment of glucagon-like peptide-1 production after roux-en-Y gastric bypass. EBioMedicine 32, 201–214. 10.1016/j.ebiom.2018.05.026 29859856 PMC6020750

[B69] Zhan M. M. Yang X. S. Zhao C. X. Han Y. H. Xie P. C. Mo Z. Q. (2024). Dietary nobiletin regulated cefuroxime- and levofloxacin-associated gut microbiota-metabolism imbalance and intestinal barrier dysfunction in mice. Food and Funct. 15, 1265–1278. 10.1039/d3fo04378a 38196314

[B70] Zhang D. D. Cheng H. Zhang Y. X. Zhou Y. C. Wu J. Liu J. (2023). Ameliorative effect of Aconite aqueous extract on diarrhea is associated with modulation of the gut microbiota and bile acid metabolism. Front. Pharmacol. 14, 1189971. 10.3389/fphar.2023.1189971 37266146 PMC10229775

[B71] Zheng X. J. Chen T. L. Jiang R. Q. Zhao A. H. Wu Q. Kuang J. L. (2021). Hyocholic acid species improve glucose homeostasis through a distinct TGR5 and FXR signaling mechanism. Cell. Metab. 33, 791–803.e7. 10.1016/j.cmet.2020.11.017 33338411

[B72] Zhou H. Liu K. Liu W. Wu M. Wang Y. Lv Y. (2023). Diets enriched in sugar, refined, or whole grain differentially influence plasma cholesterol concentrations and cholesterol metabolism pathways with concurrent changes in bile acid profile and gut microbiota composition in ApoE(-/-) mice. J. Agric. Food Chem. 71, 9738–9752. 10.1021/acs.jafc.3c00810 37307383

