# Supplementary material for: Suppressed intestinal secondary bile acids in moxifloxacin-induced hyperglycemia: studies in normal and diabetic GK rats
Source: Front Pharmacol. 2025 Apr 4;16:1569856. doi: 10.3389/fphar.2025.1569856 (PMC12006139; doi:10.3389/fphar.2025.1569856)
Supplement: Supplementary file 1 [file DataSheet1.docx]

Supplementary Material

# Supplementary Tables and Figures

## Supplementary Tables

**Table S1.** **List of the retention time and mass spectrometric parameters of 19 BAs.**

| **BAs** | **Retention time /min** | **Q1(m/z)** | **Q3(m/z)** | **DP** | **CE** |
| --- | --- | --- | --- | --- | --- |
| CA | 6.46 | 407.6 | 407.6 | -137 | -6 |
| DCA | 19.13 | 391.6 | 391.6 | -150 | -7 |
| CDCA | 18.12 | 391.7 | 391.7 | -132 | -10 |
| UDCA | 8.56 | 391.6 | 391.6 | -138 | -10 |
| LCA | 13.79 | 375.7 | 375.7 | -126 | -6 |
| CA-D4 | 11.28 | 411.4 | 411.4 | -110 | -10 |
| GCA | 5.24 | 464.7 | 464.7 | -120 | -6 |
| GDCA | 13.797 | 448.6 | 448.6 | -110 | -7 |
| GCDCA | 11.28 | 448.7 | 448.7 | -120 | -8 |
| GUDCA | 6.69 | 448.6 | 448.6 | -120 | -8 |
| GLCA | 19.47 | 432.8 | 432.8 | -125 | -8 |
| TCA | 6.13 | 514.7 | 514.7 | -160 | -8 |
| TDCA | 17.20 | 498.8 | 498.8 | -146 | -8 |
| TCDCA | 14.43 | 498.8 | 498.8 | -145 | -8 |
| TUDCA | 6.69 | 498.8 | 498.8 | -145 | -9 |
| TLCA | 19.57 | 482.6 | 482.6 | -150 | -7 |
| βMCA | 4.68 | 407.5 | 407.5 | -138 | -8 |
| TαMCA | 3.63 | 514.5 | 514.5 | -140 | -10 |
| TβMCA | 3.88 | 514.4 | 514.4 | -140 | -10 |
| αMCA | 4.25 | 407.0 | 407.0 | -160 | -15 |
| CDCA-D4 (IS) | 6.39 | 395.3 | 395.3 | -134 | -9 |

**Table S2. List of primer sequences for real-time PCR.**

| **Species** | **Gene** | **Forward primer (5’-3’)** | **Reverse primer (3’-5’)** |
| --- | --- | --- | --- |
| Rat | FXR | GCCCACTGAGAGTGTGTACC | TCCCATCTCTCTGCACTTCC |
|  | TGR5 | GCTACTGGAGTGGTAGGCAG | TCAGTCTTGGCCTATGAGCG |
|  | β-actin | CACCCGCGAGTACAACCTTC | CCCATACCCACCATCACACC |
| Human | FXR | TCTCCTGGGTCGCCTGACT | ACTGCACGTCCCAGATTTCAC |
|  | TGR5 | TCAGCCAGGACACCAGACAT | TGGGCCTTCCTGAGTGTCA |
|  | β-actin | AGCGAGCATCCCCCAAAGTT | GGGCACGAAGGCTCATCATT |

#

## Supplementary Figures


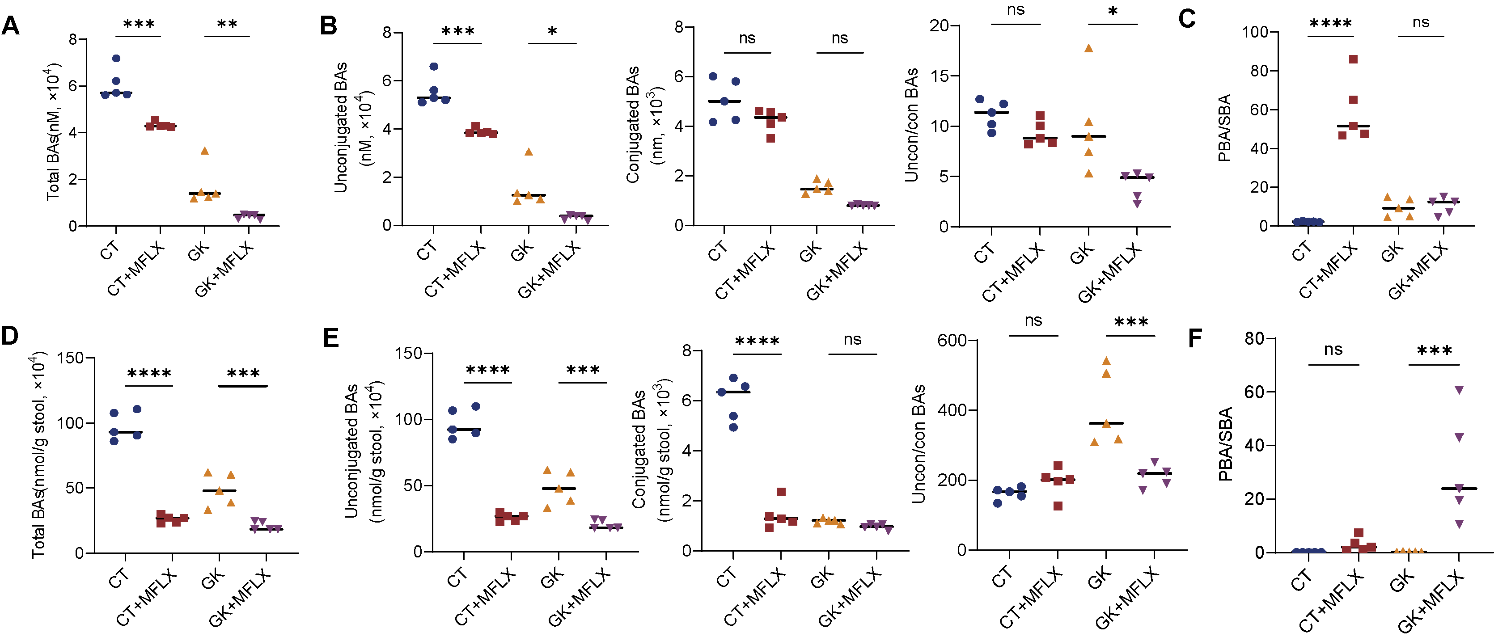


**Supplementary Figure 1.** Dynamic change of BAs profile in serum and feces after MFLX treatment for 3 days**. (A, D)** Total BAs levels in serum **(A)** and feces **(D)** following 3 days of MFLX treatment; **(B, E)** The levels of unconjugated BAs, conjugate BAs and Uncon/con in serum **(B)** and feces **(E)**; **(C, F)** PBA/SBA in serum **(C)** and feces **(F)**. Data are expressed as mean ± SD (n = 5). **p* < 0.05, ***p* < 0.01, ****p* < 0.001, *****p* < 0.0001.


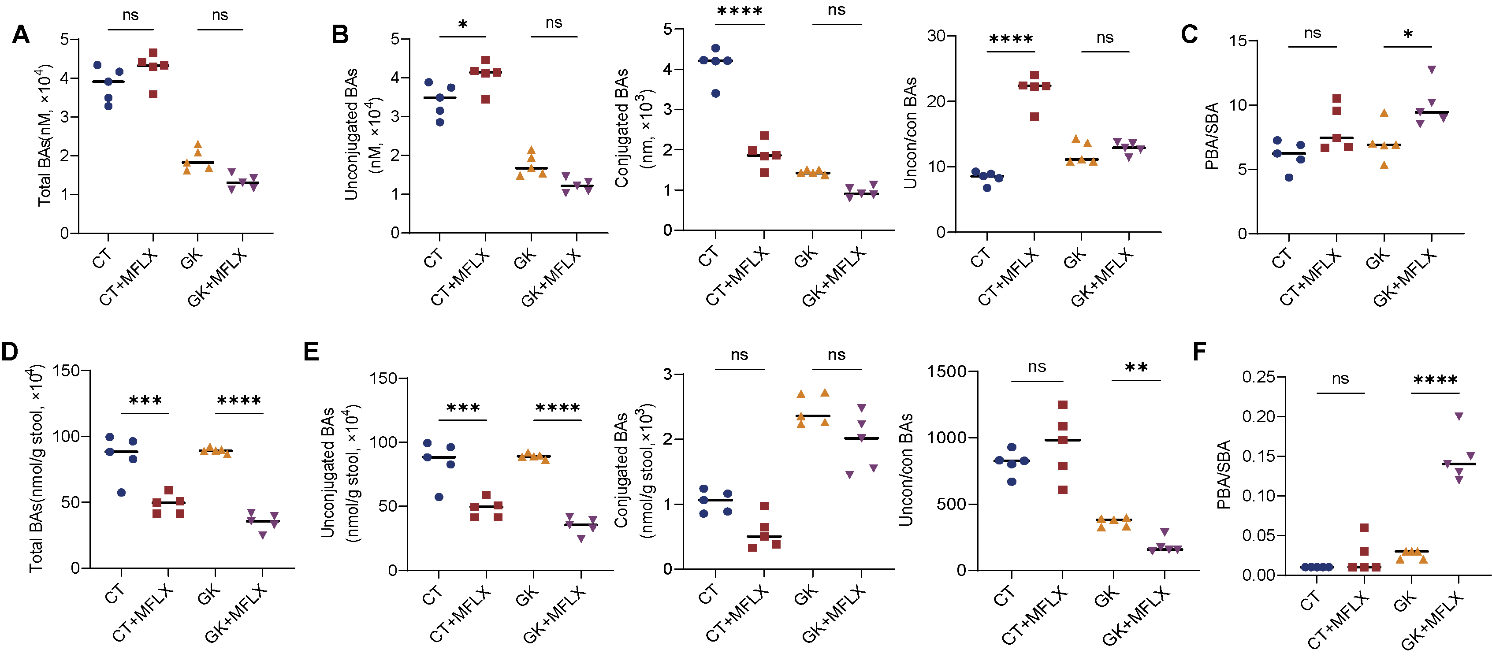


**Supplementary Figure 2.** Dynamic change of BAs profile in serum and feces after MFLX treatment for 3 days**. (A, D)** Total BAs levels in serum **(A)** and feces **(D)** following 14 days of MFLX treatment; **(B, E)** The levels of unconjugated BAs, conjugate BAs and Uncon/con in serum **(B)** and feces **(E)**; **(C, F)** PBA/SBA in serum **(C)** and feces **(F)**. Data are expressed as mean ± SD (n = 5). **p* < 0.05, ***p* < 0.01, ****p* < 0.001, *****p* < 0.0001.


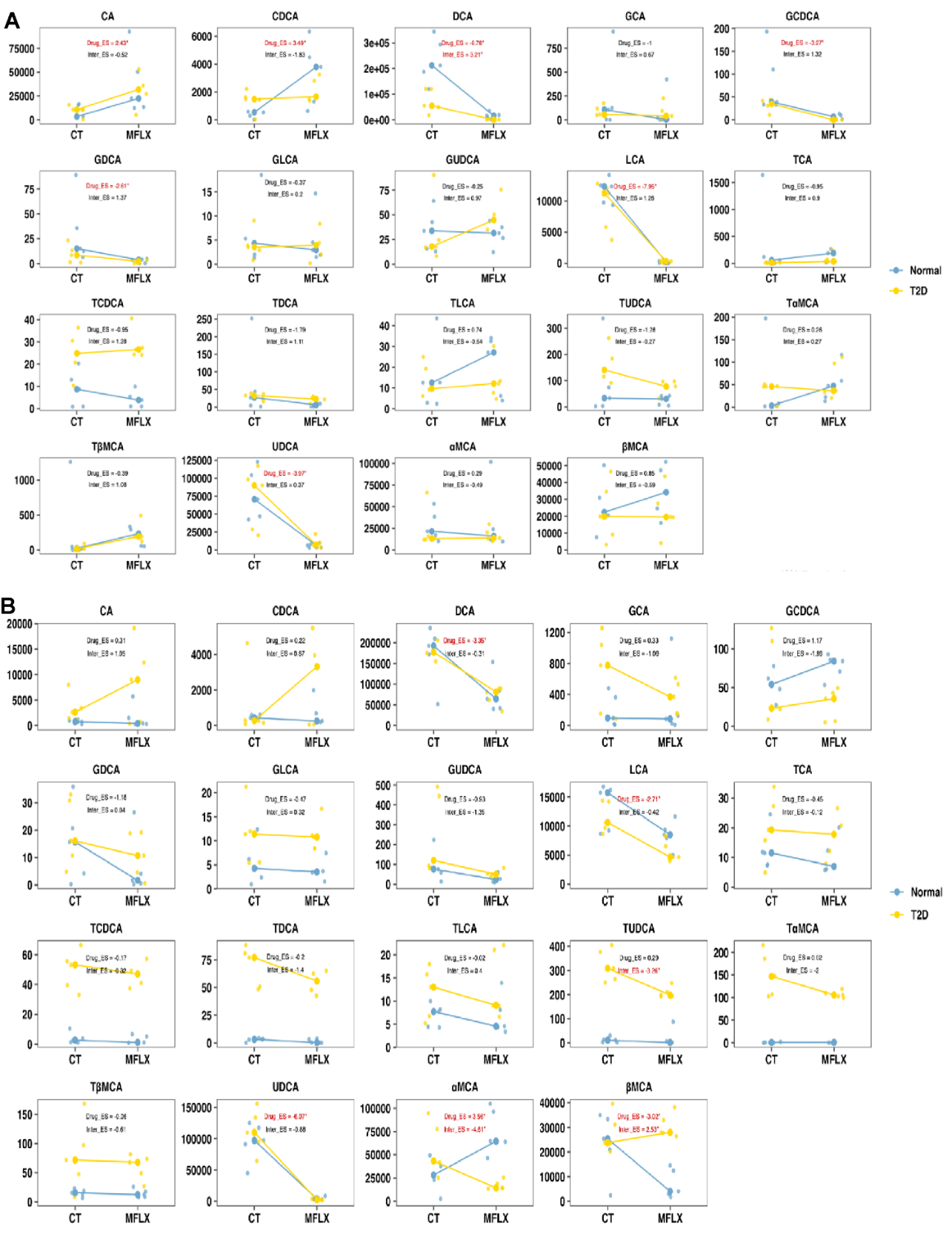


**Supplementary Figure 3.** Alterations in intestinal SBAs levels. **(A, B)** Linear regression model incorporating interaction terms model of 19 BAs after MFLX treatment for 3 days **(A)** and 14 days **(B)**. The model included a main effect of disease, a main effect of drug interaction effect, an interaction effect between drug and disease. Non-parallel lines suggest a differential drug effect across disease states, where a widening angle indicates an amplification of differences and a narrowing angle implies a reduction in differences.


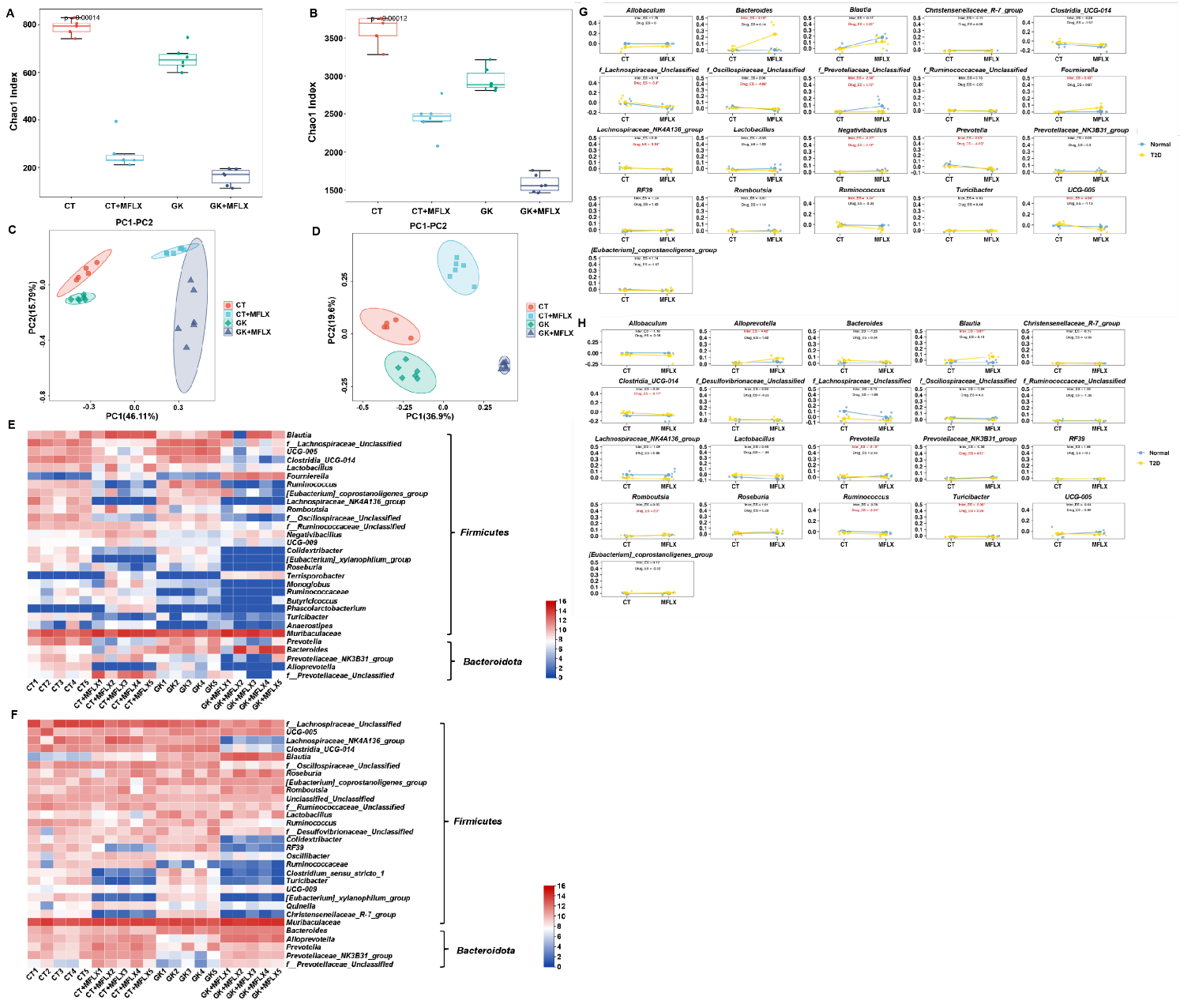


**Supplementary Figure 4.** MFLX-induced changes in gut microbiota. **(A, B)** α-diversity of the gut microbiota, as indicated by the Chao indices and observed species following MFLX administration for 3 days **(A)** and 14 days **(B)**; **(C, D)** Principal coordinate analysis (PCoA) plot generated using OTU metrics based on the Binary-Jaccard similarity for the four groups after 3 days **(C)** and 14 days **(D)** of administration; **(E, F)** Heatmaps of the relative abundance of the top 31 genera; **(G, H)** Linear regression model incorporating interaction terms model of genera after MFLX treatment for 3 days **(G)** and 14 days **(H)**.
